# Epigenetic priming targets tumor heterogeneity to shift transcriptomic phenotype of pancreatic ductal adenocarcinoma towards a Vitamin D susceptible state

**DOI:** 10.1038/s41419-024-06460-9

**Published:** 2024-01-26

**Authors:** Bo He, Lauren Stoffel, Clifford Jiajun He, Kumsun Cho, Albert M. Li, Haowen Jiang, Brittany M. Flowers, Kha The Nguyen, Kelly Wen Wang, Audrey Yixin Zhao, Meng-Ning Zhou, Sofia Ferreira, Laura D. Attardi, Jiangbin Ye

**Affiliations:** 1 Department of Radiation Oncology, Stanford University School of Medicine, Stanford, CA 94305 USA; 2 Cancer Biology Program, Stanford University School of Medicine, Stanford, CA 94305 USA; 3 Stanford Cancer Institute, Stanford University School of Medicine, Stanford, CA 94305 USA; 4 Department of Genetics, Stanford University School of Medicine, Stanford, CA 94305 USA

**Keywords:** Tumour heterogeneity, Pancreatic cancer

## Abstract

As a highly heterogeneous tumor, pancreatic ductal adenocarcinoma (PDAC) exhibits non-uniform responses to therapies across subtypes. Overcoming therapeutic resistance stemming from this heterogeneity remains a significant challenge. Here, we report that Vitamin D-resistant PDAC cells hijacked Vitamin D signaling to promote tumor progression, whereas epigenetic priming with glyceryl triacetate (GTA) and 5-Aza-2′-deoxycytidine (5-Aza) overcame Vitamin D resistance and shifted the transcriptomic phenotype of PDAC toward a Vitamin D-susceptible state. Increasing overall H3K27 acetylation with GTA and reducing overall DNA methylation with 5-Aza not only elevated the Vitamin D receptor (VDR) expression but also reprogrammed the Vitamin D-responsive genes. Consequently, Vitamin D inhibited cell viability and migration in the epigenetically primed PDAC cells by activating genes involved in apoptosis as well as genes involved in negative regulation of cell proliferation and migration, while the opposite effect of Vitamin D was observed in unprimed cells. Studies in genetically engineered mouse PDAC cells further validated the effects of epigenetic priming for enhancing the anti-tumor activity of Vitamin D. Using gain- and loss-of-function experiments, we further demonstrated that VDR expression was necessary but not sufficient for activating the favorable transcriptomic phenotype in respond to Vitamin D treatment in PDAC, highlighting that both the VDR and Vitamin D-responsive genes were prerequisites for Vitamin D response. These data reveal a previously undefined mechanism in which epigenetic state orchestrates the expression of both VDR and Vitamin D-responsive genes and determines the therapeutic response to Vitamin D in PDAC.

## Introduction

Pancreatic ductal adenocarcinoma (PDAC) is a highly lethal cancer that is projected to become the second leading cause of cancer-related death in the United States in the next ten years [1, 2]. Moreover, the 5-year survival rate of patients with PDAC at the time of diagnosis has remained stagnant since the 1960s [3, 4], largely due to resistance to a broad spectrum of therapies. A better understanding of widely occurring drug resistance is vital for improving survival rates in PDAC patients.

Vitamin D and its analogs, with their ability to regulate cell growth, differentiation, apoptosis, and angiogenesis, have been widely considered as a promising therapeutic agent for cancers, including pancreatic cancer [5, 6]. Although compelling evidence supports Vitamin D’s beneficial effects on reshaping the tumor microenvironment through reprograming the cancer-associated fibroblasts [5, 7–9], the effects of Vitamin D on tumor cells are contradictory. For example, Vitamin D has favorable effects in some PDAC cell lines or specimens, but has no or even unfavorable effects, in others. Although the first observations of the inconsistency of the Vitamin D response in different PDAC cell lines date to the 1990s [10–13], the underlying mechanisms remain largely unknown. The most recent study in human PDAC cells with defined subtypes showed that Vitamin D increased metastatic propensity in basal-like PDAC cells, but had inverse effects on classical subtypes, suggesting that PDAC responds to Vitamin D treatment in a subtype-dependent manner [14]. Consistently, Vitamin D receptor (VDR) expression also exhibits subtype preference with higher expression in the classical subtypes but lower expression in the basal-like subtypes [10, 14]. As a cancer known for its high heterogeneity and notable plasticity, PDAC cells resistant to Vitamin D could be preferentially selected during treatment. These cells not only exhibit resistance to Vitamin D therapy but can also hijack Vitamin D signaling pathways to further cancer progression, leading to a more aggressive malignancy [14]. This may explain why the therapeutic efficacy of Vitamin D has not met expectations, in spite of its beneficial effects in reprogramming cancer-associated fibroblasts for improving drug delivery [7, 9].

Increasing evidence suggests that phenotypic heterogeneity in PDAC is hierarchically controlled through epigenetic regulations [15]. Epigenetic reprogramming has therefore been proposed as an appealing strategy for targeting tumor heterogeneity and switching transcriptomic phenotype toward a drug susceptible state in PDAC [16–19]. As an emerging concept in PDAC treatment, epigenetic treatment is still in its infancy, since only a few epigenetic drugs, including histone deacetylase (HDAC) inhibitors and DNA methyltransferase (DNMT) inhibitors, qualify for use in clinical trials [17, 20]. Preclinical studies have shown that HDAC inhibitors and DNMT inhibitors, which target histone hypo-acetylation and DNA hyper-methylation respectively, are capable of reprogramming PDAC cells and inhibiting tumor growth in mice [21, 22]. Thus, we postulated that epigenetic treatment strategies might be employed to shift the molecular and cellular heterogeneities of PDAC toward a Vitamin D-susceptible state so that we can maximize the beneficial effects and eliminate the adverse effects of Vitamin D in PDAC treatment.

In the present study, we used glyceryl triacetate (GTA) and 5-Aza-2′-deoxycytidine (5-Aza), which target histone hypo-acetylation and DNA hyper-methylation, respectively, to remodel the epigenome of PDAC cells. Our results showed that epigenetic priming upregulates VDR expression and reprograms Vitamin D-responsive genes by increasing H3K27ac with GTA and decreasing DNA methylation with 5-Aza, thereby shifting PDAC cells toward a Vitamin D-susceptible state. Together with the finding that both the VDR and Vitamin D-responsive genes are prerequisites for the anti-tumor activity of Vitamin D, we propose a previously undefined mechanism in which the epigenetic state plays a central role in controlling the expression of both VDR and Vitamin D-responsive genes to determine the “Yin and Yang” effects of Vitamin D treatment in PDAC.

## Results

### Increasing global H3K27ac with GTA elevates VDR expression and enhances anti-proliferative activity of Vitamin D in Vitamin D-resistant human PDAC cells

To test the hypothesis that epigenetic priming could shift PDAC cells from a Vitamin D resistant state to a Vitamin D-susceptible state, we investigated two well-known Vitamin D resistant PDAC cell lines, MiaPaca2 and Panc1, and one Vitamin D-sensitive PDAC cell line, BxPC3. Consistent with a previous report [10], MiaPaca2 and Panc1 cells are resistant to Vitamin D analogs calcipotriol (CPT) and EB1089, whereas BxPC3 cells are sensitive to these two analogs (Fig. S1A). Given that prior studies have found a lack of VDR expression in MiaPaca2 and Panc1 cells [10, 14], we also assessed VDR expression in these cell lines. Immunoblot results showed that VDR protein is expressed in both cytosol and nucleus of BxPC3 cells, and Vitamin D analogs induce the translocation of VDR to the nucleus (Fig. S1B). In contrast, VDR levels were low to undetectable in both cytosol and nucleus of MiaPaca2 and Panc1 cells, and Vitamin D analogs only slightly increased the nuclear VDR level in these cell lines (Fig. S1B). Since both Vitamin D analogs exhibited identical effects on cell viability and VDR expression, we only used CPT in following experiments. The qRT-PCR experiments further showed that MiaPaca2 and Panc1 cells expressed significantly lower levels of *vdr* mRNA than BxPC3 cells (Fig. S1C), suggesting that VDR was silenced at transcription level.

Since the transcriptomic phenotype is hierarchically clustered and controlled by epigenetic cues [23], we postulated that the heterogeneity of VDR expression is controlled by epigenetic modifications. As mentioned above, histone hypo-acetylation and DNA hyper-methylation, which are targeted by HDAC inhibitors and DNMT inhibitors respectively, are the two major epigenetic targets being investigated in clinical trials of PDAC. Among the histone acetylation modifications, H3K27ac is the principal modification associated with chromosome accessibility and gene transcription [24]. Thus, we first explored H3K27ac occupancy in the gene region of *vdr* via published H3K27ac ChIP-Seq data in BxPC3, MiaPaca2, and Panc1 cells [25]. In line with the mRNA levels of *vdr*, a lack of H3K27ac occupancy in the promoter region of the *vdr* gene were observed in the MiaPaca2 and Panc1 cells relative to the BxPC3 cells (Fig. S2A), suggesting that H3K27 hypo-acetylation may be one cause of VDR silencing in MiaPaca2 and Panc1 cells. To test this hypothesis, we treated MiaPaca2 and Panc1 cells with suberoylanilide hydroxamic acid (SAHA), an HDAC inhibitor that has been reported to increase global H3K27ac levels [26]. As expected, SAHA not only increased global H3K27ac levels and VDR protein levels, but also slightly sensitized MiaPaca2 and Panc1 cells to Vitamin D treatment (Fig. S2B, C). Despite SAHA alone partially restoring VDR expression and sensitizing Vitamin D response, combination treatment with 5-Aza was toxic enough to mask the effects of Vitamin D (Fig. S2C). Thus, we sought to explore a less toxic strategy for increasing global histone acetylation levels.

**Fig. 1:**
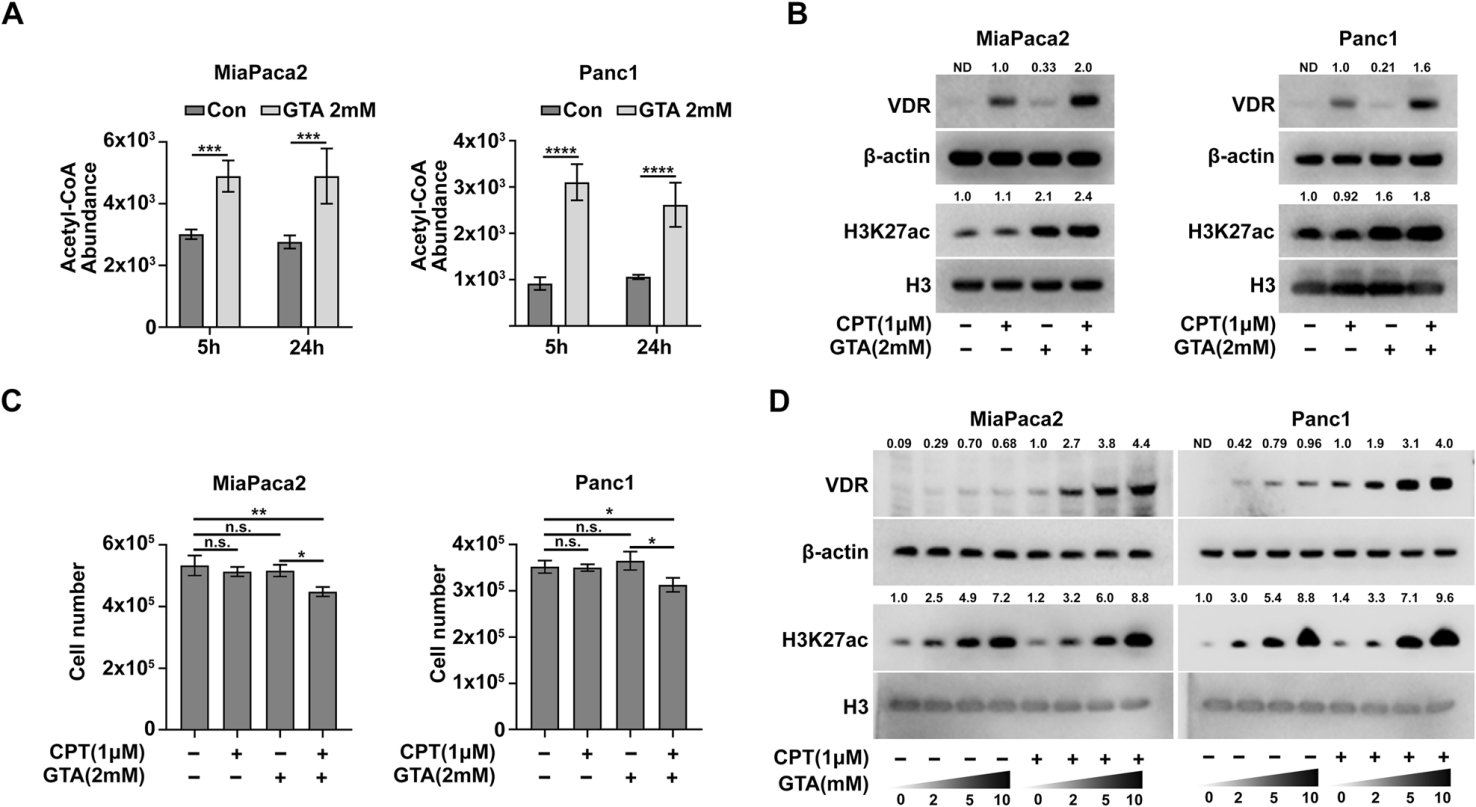
GTA elevates global H3K27ac and VDR expression in human PDAC cells. **A** LC–MS analysis showed that GTA increases intracellular acetyl-CoA levels in both MiaPaca2 and Panc1 cells. **B** Immunoblots for whole cell lysates showed that GTA elevates global H3K27ac and VDR levels in MiaPaca2 and Panc1 cells. **C** 72-hour proliferation assay of MiaPaca2 and Panc1 cells with indicated treatment. **D** Immunoblots for whole cell lysates showed that GTA elevates global H3K27ac and VDR levels in a dose-dependent manner. ND not detected or not determined. All data are plotted as Mean ± SEM. Statistical significance was determined by two-way ANOVA with Sidak’s multiple comparisons test for (**A**) and one-way ANOVA with Tukey’s multiple comparisons test for (**C**). n.s. no significance or *P* > 0.05; *P < 0.05; **P < 0.01; ***P < 0.005 and ****P < 0.001.

We previously reported that GTA, an acetate precursor that increases intracellular acetyl-CoA and global H3K27ac levels, can restore chromosome accessibility in neuroblastoma cells [27]. As an FDA approved drug, GTA is safe to use, even at high concentrations. Consistent with the results obtained in neuroblastoma cells, GTA supplementation significantly increased intracellular acetyl-CoA and global H3K27ac levels in MiaPaca2 and Panc1 cells (Fig. 1A, B). More specifically, ChIP-qPCR result shows that GTA supplementation significantly increases the H3K27ac level at the promoter region of the *vdr* gene (Fig. S2D). Along with increased H3K27ac, both the mRNA and protein levels of VDR were elevated by GTA (Fig. S3 and Fig. 1B). Because GTA treatment increases intracellular acetyl-CoA levels, it may cause a global increase in protein lysine acetylation. Notably, of all the proteins with lysine acetylation evaluated, H3, particularly H3K27ac, showed the most significant upregulation by GTA (Fig. S2E). This is consistent with our previous data that GTA is efficient in increasing H3K27ac and chromosome accessibility in both neuroblastoma and breast cancer cells [27, 28]. The cell viability assay showed that GTA treatment slightly increased the Vitamin D sensitivity in both MiaPaca2 and Panc1 cells, which is consistent with that observed for SAHA treatment (Fig. 1C and Fig. S2C). As expected, no obvious toxicity was observed in the GTA treatment group (Fig. 1C). In line with the increased global H3K27ac level, GTA increased VDR protein levels in a dose-dependent manner, especially when combined with CPT treatment (Fig. 1D). Collectively, these results suggest that VDR was silenced partially through H3K27 hypo-acetylation, and that increasing H3K27ac with SAHA or GTA elevated VDR expression and slightly enhanced the anti-proliferative activity of Vitamin D in the Vitamin D-resistant PDAC cells.

### Reducing DNA methylation with 5-Aza elevates VDR expression and enhances anti-proliferative activity of Vitamin D in Vitamin D-resistant human PDAC cells

**Fig. 2:**
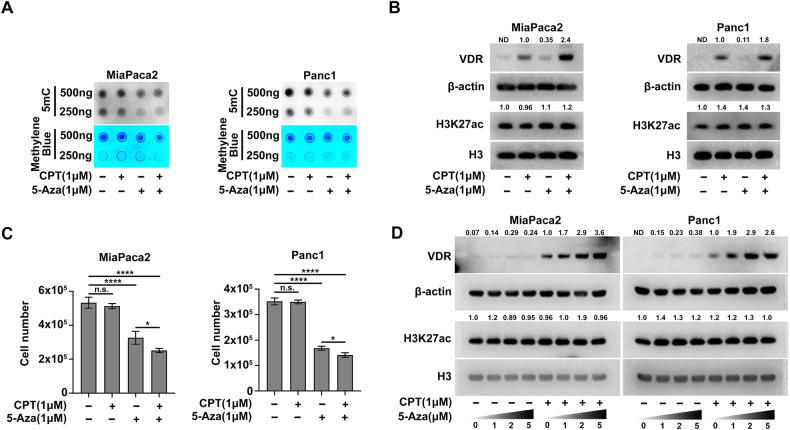
5-Aza elevates VDR expression by decreasing global DNA methylation in human PDAC cells. **A** Dot blot analysis for spotting of total DNA stained with anti-5mC (Upper) and methylene blue (Bottom) showed that 5-Aza decreases global DNA methylation in MiaPaca2 and Panc1 cells. **B** Immunoblots for whole cell lysates showed that 5-Aza elevates VDR expression irrespective of H3K27ac levels in MiaPaca2 and Panc1 cells. **C** 72-hour proliferation assay of MiaPaca2 and Panc1 cells with indicated treatment. Data are plotted as Mean ± SEM. Statistical significance was determined by one-way ANOVA with Tukey’s multiple comparisons test. n.s. no significance or *P* > 0.05; *P < 0.05 and ****P < 0.001. **D** Immunoblots for whole cell lysates showed that 5-Aza elevates VDR expression in a dose-dependent manner in MiaPaca2 rather than Panc1 cells. ND not detected or not determined.

As a key repressive epigenetic modification, DNA methylation is another target for epigenetic therapy in PDAC and is reported to be associated with tumor heterogeneity in PDAC [15, 23]. Therefore, we investigated whether DNA methylation also contributes to VDR silencing in PDAC. Analysis of published dataset [29] revealed a greater than 20-fold elevation of DNA methylation levels at the 500 bp upstream of the transcription start site (TSS) (L1, cg06369854) of the *vdr* gene in MiaPaca2 cells compared to BxPC3 cells, with a modest decrease at the TSS (L2, cg27537561) (Fig. S2F). In Panc1 cells, both L1 and L2 sites exhibited increased DNA methylation levels relative to BxPC3 cells (Fig. S2F). Analysis of the DNA methylation profile from the PDAC patient samples [30] revealed comparable DNA methylation levels in the promoter region of *vdr* (L1, cg06369854; L2, cg03137447; L3, cg27537561) between the cancer tissues and non-cancerous normal tissues. However, higher DNA methylation levels were observed in the gene body (L4, cg10592901) and 3′region (L5, cg14854850) of *vdr* gene in the cancer tissue (Fig. S2H). In addition, data from UALCAN shows that VDR expression does not exhibit significant differences between normal and cancer tissues (Fig. S2I). This lack of significant difference on methylation in the promoter region of *vdr* between cancer tissues and non-cancerous normal tissues may be attributed to the high heterogeneity of PDAC. The increase in DNA methylation at the *vdr* gene inspired us to hypothesize that *vdr* was silenced in part due to DNA hyper-methylation in the Vitamin D-resistant human PDAC cells. To test this hypothesis, we explored whether 5-Aza, a DNMT inhibitor that reduces DNA methylation in the proliferating cells [17, 31], could increase the expression of VDR. Indeed, 5-Aza treatment significantly decreased total DNA methylation in MiaPaca2 and Panc1 cells (Fig. 2A, B). The MeDIP-qPCR results further indicated that 5-Aza treatment markedly reduced the levels of DNA methylation within the promoter region of the *vdr* gene (Fig. S2G). In parallel, both mRNA levels and protein levels of VDR were increased by 5-Aza treatment (Fig. S3 and Fig. 2B). Despite a high degree of cell toxicity associated with 5-Aza treatment, it slightly enhanced the anti-proliferative activity of Vitamin D in both cell lines (Fig. 2C and Fig. S9A). In addition, a dose-dependent VDR induction with 5-Aza was observed in MiaPaca2 cells and Panc1 cells (Fig. 2D). Taken together, these results suggest that 5-Aza treatment increases VDR expression by reducing DNA methylation.

### GTA and 5-Aza synergistically elevate VDR expression and enhance the anti-proliferative activity of Vitamin D in human PDAC cells

**Fig. 3:**
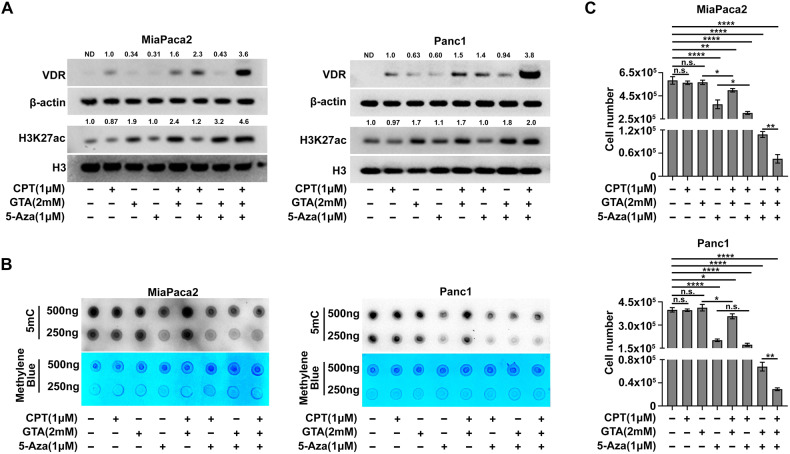
GTA and 5-Aza synergistically elevate VDR expression and sensitize human PDAC cells to Vitamin D treatment. **A** Immunoblots for whole cell lysates showed that epigenetic priming elevates H3K27ac and VDR in MiaPaca2 and Panc1 cells, ND: not detected or not determined. **B** Dot blot analysis for spotting of total DNA stained with anti-5mC (Upper) and methylene blue (Bottom) in MiaPaca2 and Panc1 cells with indicated treatments. **C** Cell viability assay of MiaPaca2 and Panc1 cells with indicated treatment. Data are plotted as Mean ± SEM. Statistical significance was determined by one-way ANOVA with Tukey’s multiple comparisons test. n.s. no significance or *P* > 0.05; *P < 0.05; **P < 0.01 and ****P < 0.001.

Since both GTA and 5-Aza treatment elevated VDR expression and enhanced anti-proliferative activity of CPT while targeting different epigenetic modifications (Figs. 1 and 2), we hypothesized that GTA and 5-Aza have synergistic effects in overcoming Vitamin D resistance in PDAC cells. To test this hypothesis, we conducted a combination treatment experiment. Compared to GTA or 5-Aza treatment alone, GTA and 5Aza combination treatment simultaneously elevated H3K27ac and decreased DNA methylation (Fig. 3A, B). Importantly, combination treatment with GTA and 5-Aza increased both mRNA and protein levels of VDR expression more significantly than either GTA or 5-Aza treatment alone (Fig. 3A and Fig. S3). Unlike GTA and 5-Aza, CPT treatment did not directly increase mRNA level of *vdr* in any of the three human PDAC cells (Fig. S1C and Fig. S3). However, CPT treatment did increase the protein level of VDR and translocation of VDR into nuclear (Fig. S1B and Fig. 3A). These results suggest that Vitamin D primarily functions on stabilizing the VDR protein and translocating the VDR into nuclear to regulate the downstream Vitamin D responsive genes, which is consistent with previous reports [32–34]. Although the epigenetic priming with GTA and 5-Aza showed strong inhibitory effects on cell viability, CPT supplementation further decrease the cell viability of the epigenetic primed cells (Fig. 3C), suggesting that epigenetic priming with GTA and 5-Aza is efficient to shift the PDAC cells towards a Vitamin D susceptible state. Increasing the concentration of either GTA or 5-Aza in the combination treatment did not further enhance the anti-tumor activity of CPT (Fig. S4A, B). We speculate that the enhanced cell toxicity at higher concentrations of GTA or 5-Aza may mask the anti-tumor activity of Vitamin D. Since 5-Aza treatment alone exhibits high cell toxicity, we also tested whether combination treatment with a lower concentration of 5-Aza would maximize the effects of Vitamin D. However, combination treatment with a lower concentration of 5-Aza did not show a more pronounced effect in enhancing the anti-proliferative activity of Vitamin D, despite the reduced toxicity to PDAC cells (Fig. S4C). Together, these data suggest that increasing H3K27ac with GTA and decreasing DNA methylation with 5-Aza synergistically elevate VDR expression and enhance the anti-tumor activity of Vitamin D in PDAC cells, even though the combination treatment with GTA and 5-Aza has a high degree of cell toxicity.

### Epigenetic priming with GTA and 5-Aza reprograms Vitamin D-responsive genes to activate the anti-tumor activity of Vitamin D

**Fig. 4:**
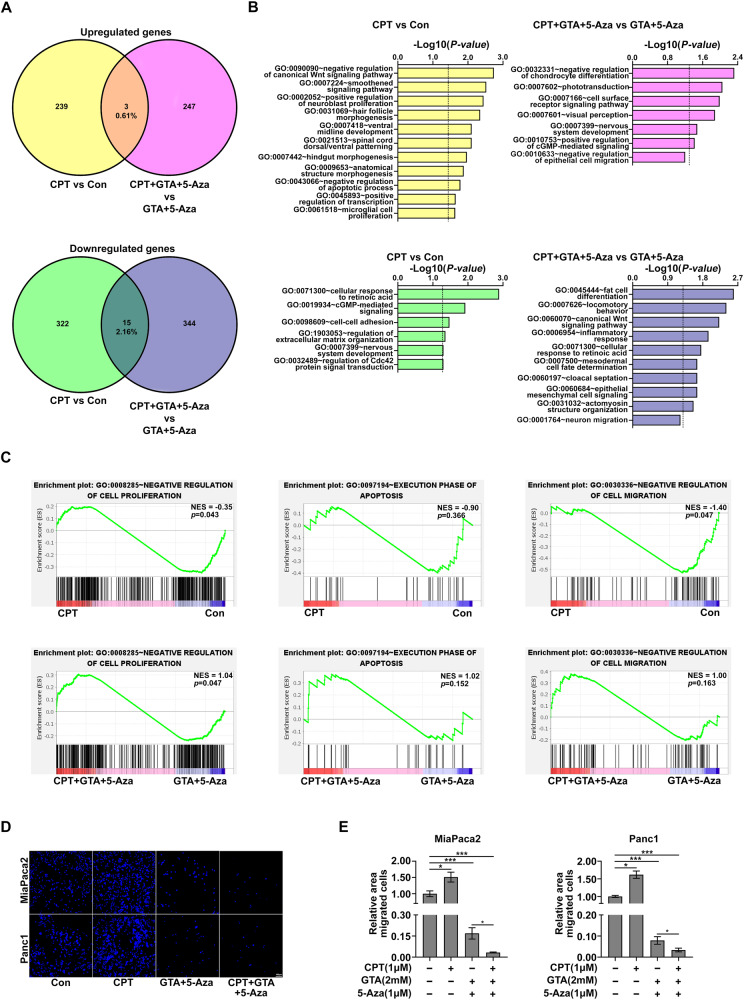
Epigenetic priming with GTA and 5-Aza reprograms Vitamin D-responsive genes to shift human PDAC cells toward a Vitamin D-susceptible state. **A** Venn diagrams show the number of the up-regulated and down-regulated Vitamin D-responsive genes in control and epigenetically primed MiaPaca2 cells. Intersections represent the number of Vitamin D-responsive genes with consensus expression pattern. **B** The bar chart shows the GO analysis of up-regulated and down-regulated Vitamin D-responsive genes in unprimed and epigenetically primed MiaPaca2 cells. The dashed line indicates that *P* = 0.05. **C** GSEA analysis of negative regulators of cell proliferation, apoptosis genes and negative regulators of cell migration by comparing Vitamin D with DMSO treatment in unprimed (Upper) and epigenetically primed (Bottom) MiaPaca2 cells. **D** Representative images show the migration of pretreated PDAC cells across 8-μm filters. The nuclei were stained blue with 4′, 6-diamidino-2-phenylindole (DAPI). (Scale bar, 100 μM). **E** Quantification of area covered by DAPI stained cells as shown in (**D**). Data are plotted as Mean ± SEM. Statistical significance was determined by one-way ANOVA with Tukey’s multiple comparisons test for (**C**). *P < 0.05 and ***P < 0.005.

To further elucidate the underlying mechanisms by which epigenetic priming with GTA and 5Aza enhances anti-proliferative activity of Vitamin D in PDAC, RNA-seq was performed in both unprimed and epigenetically primed MiaPaca2 cells treated with DMSO or CPT, respectively. Differential expression analysis demonstrated that three-fold more genes were up-regulated by epigenetically priming with GTA and 5-Aza than the unprimed control (Fig. S5A), confirming that increasing global H3K27ac and decreasing global DNA methylation activate gene transcription. CPT treatment does not affect this transcriptional activation (Fig. S5B–D). Among the differentially expressed genes (DEGs), tumor suppressor genes for PDAC, such as CDKN1A (or p21), DUSP10, and NOG, were significantly upregulated, whereas the oncogenes, such as DOCK1, EFNA1, Hey1, and TGFB2, were significantly downregulated (Fig. S5A). To further understand how epigenetic priming elevates these tumor suppressor genes, we analyzed the epigenetic states of the upregulated genes via the published datasets and observed that these genes are characterized with hypo-H3K27ac and hyper-DNA methylation (Figs. S6A and S7A). Epigenetic priming with GTA and 5-Aza significantly elevate H3K27ac level and decrease DNA methylation level at the promoter regions of these genes (Figs. S6B and S7B), suggesting that these tumor suppressor genes are epigenetically silenced in MiaPaca2 cells. In addition, GO enrichment analysis of the DEGs revealed that pathways involved in cell differentiation, negative regulation of cell proliferation and migration as well as positive regulation of apoptosis were significantly enriched in the upregulated genes. In contrast, pathways related epithelial to mesenchymal transition and positive regulation of epithelial cell migration were significantly enriched in downregulated genes, although these pathways are not within the top 10 pathways (Fig. S8A). Gene set enrichment analysis (GSEA) further showed that genes related to cell death, cell apoptosis, cell-cell adhesion, and negative regulation of cell proliferation and migration, were generally upregulated by epigenetic priming (Fig. S8B, C). The transcriptional signatures are in line with the observation that epigenetic priming not only decreased cell proliferation and migration but also increased cell death (Fig 3C, Fig. S9A, Fig 4D, E). Compared to epigenetic primming with GTA and 5-Aza, the addition of CPT has obvious beneficial effects on inhibiting cell migration and proliferation, which is supported by the more significant enrichment, as indicated by smaller *P* value, observed in the “negative regulation of cell migration”, “negative regulation of cell proliferation”, “cell differentiation” and “epithelial cell differentiation” pathways among the upregulated genes as well as the “positive regulation of cell migration” pathway among the downregulated genes (Fig. S8D). Furthermore, the addition of CPT further activates pathways associated with “negative regulation of viral genome replication” and “response to interferon-beta”, but represses pathways involved in “homophilic cell adhesion via plasma membrane adhesion molecules” and “regulation of GTPase activity” (Fig. S8D), implying that the CPT addition under the epigenetic priming condition may regulate the tumor immunological response. Together, these results suggest that epigenetic priming with GTA and 5-Aza comprehensively reprograms the transcriptional signature and demonstrates strong anti-tumor activity in PDAC cells. The addition of CPT to the combination treatment with GTA and 5-Aza further enhances the anti-tumor effects in PDAC.

Next, we analyzed the DEGs induced by CPT in unprimed or epigenetically primed MiaPaca2 cells. Interestingly, CPT treatment significantly upregulated SHH, an oncogene that promotes the progression and metastasis in PDAC [35, 36], but downregulated CCNG2, a key tumor suppressor gene in PDAC [37, 38] (Fig. S5E), suggesting a pro-tumor activity of Vitamin D in MiaPaca2 cells without epigenetic priming. However, in the epigenetically primed cells, CPT treatment significantly upregulated MT1G, a reported Vitamin D responsive gene [39] and tumor suppressor gene for PDAC [40], that exhibits epigenetic characters with hyper DNA methylation in the promoter region (Fig. S7A), but downregulated RAC2, a Small GTPase referred to as the Ras proto-oncogene superfamily [41] (Fig. S5F). Epigenetic priming with 5-Aza significantly reprograms the epigenome of *mt1g* through decreasing DNA methylation (Fig. S7B), which supports the idea that epigenetic state determines the responsiveness to Vitamin D treatment. Intriguingly, *mt1g* also exhibits higher DNA methylation level and lower expression level in the cancer tissue in PDAC patients (Fig. S7C, D), suggesting that epigenetic silence of *mt1g* through DNA hyper-methylation is common in PDAC cells. While a comparable number of DEGs induced by CPT were observed in both unprimed and epigenetically primed MiaPaca2 cells (Fig. S5E, F), there was minimal overlap in their DEG profiles (Fig. 4A). This was further validated by Gene Ontology (GO) analysis, which revealed distinct biological pathways being enriched in each case (Fig. 4B). Specifically, CPT treatment in unprimed cells upregulated genes involved in cell proliferation and negative regulation of apoptosis and downregulated genes involved in cell-cell adhesion. However, in epigenetically primed MiaPaca2 cells, CPT treatment upregulated genes involved in negative regulation of cell migration and downregulated the genes involved in epithelial mesenchymal cell signaling and migration (Fig. 4B). Intriguingly, GSEA analysis further demonstrated the opposite effect of CPT in regulating genes involved in cell proliferation, apoptosis, and cell migration, in which CPT treatment tended to upregulate negative regulators for cell proliferation and migration, along with apoptosis genes, in epigenetically primed cells, but tended to downregulate these genes in unprimed cells (Fig. 4C). In line with the transcriptomic alternations, results from transwell assays showed that epigenetic priming shifted the cellular response to CPT from increasing migration capacity in unprimed cells toward decreasing migration capacity in epigenetically primed cells (Fig. 4D, E). Given that epigenetic priming induced high levels of cell death (Fig. S9A, B), we speculated that the extremely low level of cell migration in the epigenetically primed cells may be partially due to cell death. Thus, we normalized migrated cells with cell survival. The normalized data further shows that CPT treatment promoted migration in unprimed cells but inhibited migration in epigenetically primed PDAC cells (Fig. S9C). The observed inverse gene expression pattern for negative regulators of cell proliferation and apoptosis genes may be the underlying mechanism by which CPT reduces cell viability in epigenetically primed PDAC cells (Fig. 3C and Fig. S9A). Collectively, these data suggest that epigenetic priming with GTA and 5-Aza reprograms Vitamin D-responsive genes such that the cellular response to Vitamin D in PDAC is shifted from pro-tumor activity toward anti-tumor activity.

### Epigenetic priming with GTA and 5-Aza enhances anti-tumor activity of Vitamin D in genetically engineered mouse PDAC cells

**Fig. 5:**
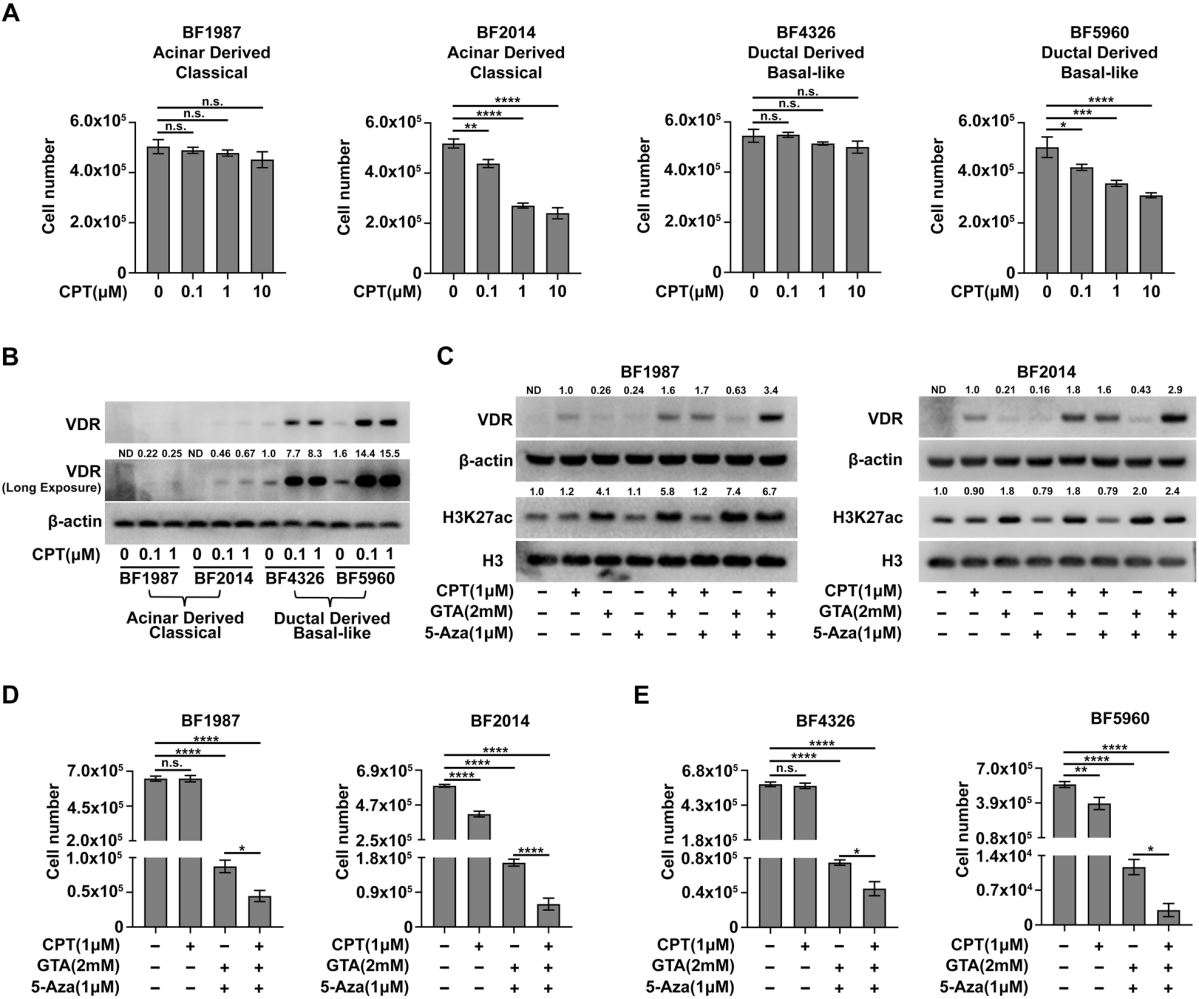
Epigenetic priming with GTA and 5-Aza enhances anti-proliferative activity of Vitamin D in genetically engineered mouse PDAC cells. **A** 72-hour proliferation assay of mouse PDAC cells treated with different concentrations of CPT. **B** Immunoblots assay of whole cell lysates showing VDR expression in mouse PDAC cells treated with different concentrations of CPT. **C** Immunoblot assay of whole cell lysates showed that epigenetic priming elevates VDR expression in VDR low-expressed mouse PDAC cells. **D**, **E** 72-hour proliferation assay of mouse PDAC cells showed that epigenetic priming with GTA and 5-Aza enhances anti-proliferative activity of CPT in mouse PDAC cells. The genotypes of the mouse PDAC cells are as follows: BF1987, *Ptf1a*^CreER/+^*Kras*^G12D/+^*P53*^fl/fl^; BF2014, *Ptf1a*^CreER/+^*Kras*^G12D/+^*P53*^fl/R172H^; BF4326, *Sox9*^CreER/+^*Kras*^G12D/+^*P53*^fl/R172H^; BF5960, *Sox9*^CreER/+^*Kras*^G12D/+^*P53*^fl/fl^. ND not detected or not determined. All data are plotted as Mean ± SEM Statistical significance was determined by one-way ANOVA with Tukey’s multiple comparisons test. n.s. no significance or *P* > 0.05; *P < 0.05; **P < 0.01; ***P < 0.005 and ****P < 0.001.

A previous study suggested that PDAC cells respond to Vitamin D treatment in a subtype-dependent manner [14], we thus questioned whether epigenetic priming enhances the anti-tumor activity of Vitamin D according to subtype preference. To test this conjecture, we expanded our research to genetically engineered mouse PDAC cells with defined subtypes and cells of origin [42]. Two acinar-derived classical cell lines (BF1987 and BF2014) and two ductal-derived basal-like cell lines (BF4326 and BF5960) were investigated. Unlike human PDAC cells, each subtype contains both Vitamin D-sensitive cells (BF2014 and BF5960) and Vitamin D-resistant cells (BF1987 and BF4326) (Fig. 5A), suggesting that Vitamin D response in mouse PDAC cells is not subtype-dependent. VDR expression showed obvious subtype preferences, but the pattern was opposite to that found in human PDAC cells [14], as the two classical mouse PDAC cell lines have consistently low levels of VDR, whereas the two basal-like cell lines have consistently high levels of VDR (Fig. 5B). The finding that cellular sensitivity to Vitamin D does not consistently align with VDR expression levels (Fig. 5A, B) suggests that VDR expression is not the sole determinant of Vitamin D’s anti-tumor efficacy.

Consistent with human PDAC cells, epigenetic priming with GTA and 5-Aza elevated VDR expression in the VDR low-expressed mouse PDAC cells (Fig. 5C), suggesting that the mechanism of VDR silencing is conserved between mouse and human PDAC cells. Intriguingly, epigenetic priming enhanced anti-proliferative activity of Vitamin D in all mouse PDAC cells, irrespective of the original Vitamin D response and VDR expression levels (Fig. 5D, E). Consistently, epigenetic priming also further enhanced the anti-proliferative activity of Vitamin D in BxPC3 cells (Fig. S10A), the Vitamin D-sensitive human PDAC cell line with high VDR expression. Moreover, combination treatment showed less toxicity in the untransformed cells, including 293 T cells and primary MEF cells, than in the PDAC cells (Fig. S10B, C). These data collectively suggest that epigenetic priming with GTA and 5-Aza enhances anti-proliferative activity of Vitamin D in PDAC cells, regardless of their original response or VDR expression levels. These findings also hold potential clinical significance in guiding ongoing clinical trials of Vitamin D in PDAC.

### VDR is necessary for augmenting the original Vitamin D response but insufficient for shifting PDAC cells toward a Vitamin D-susceptible state

**Fig. 6:**
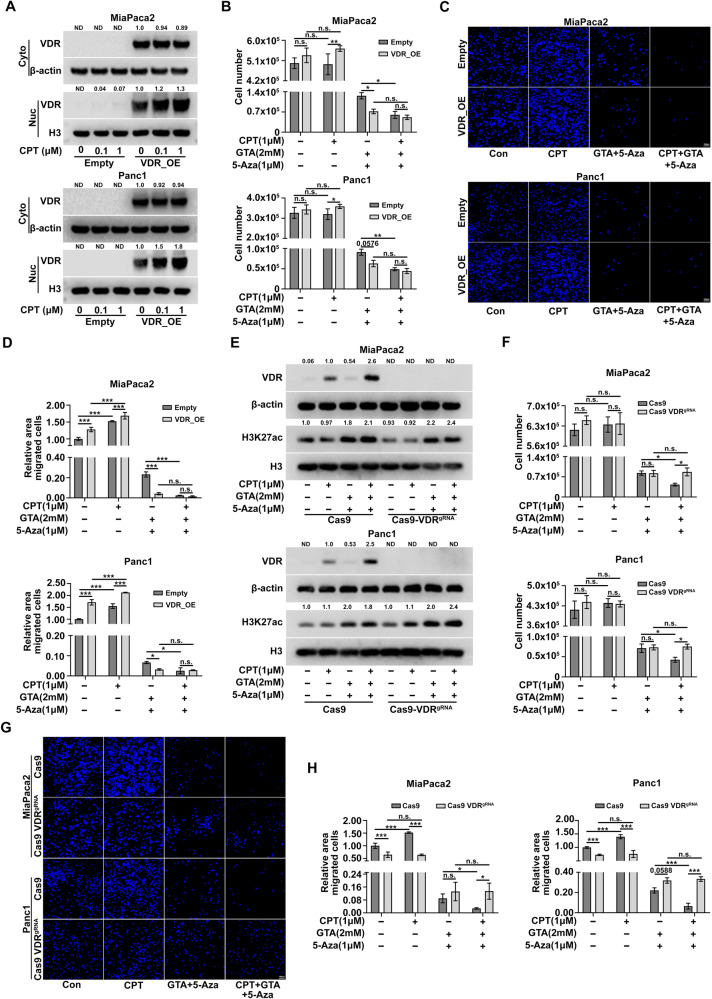
The VDR is necessary but not sufficient for the anti-tumor activity of Vitamin D in human PDAC cells. **A** VDR overexpression was determined by immunoblot analysis of cytosolic and nuclear lysates from pCDH vector control (pCDH-empty) or pCDH-VDR transfected PDAC cells with different concentrations of CPT treatment. **B** 72-hour proliferation assay of transfected PDAC cells with indicated treatment. **C** Representative images for the migration of pCDH-empty and pCDH-VDR transfected PDAC cells pretreated as indicated. **D** Quantification of area covered by DAPI stained cells as shown in (**C**). **E** VDR knock out efficiency was determined by immunoblot analysis of whole cell lysates from Cas9 and Cas9-VDR^gRNA^ transfected PDAC cells with indicated treatment. **F** 72-hour proliferation assay of Cas9 and Cas9-VDR^gRNA^ transfected PDAC cells with indicated treatment. **G** Representative images for the migration of Cas9 and Cas9-VDR^gRNA^ transfected PDAC cells pretreated as indicated. **H** Quantification of area covered by DAPI stained cells as shown in **G**. ND not detected or not determined. All data are plotted as Mean ± SEM. Statistical significance was determined by two-way ANOVA with Sidak’s multiple comparisons test. n.s. no significance or *P* > 0.05; *P < 0.05; **P < 0.01; ***P < 0.005 and ****P < 0.001.

The dissociation between VDR expression levels and Vitamin D response led us to investigate the role of VDR in the anti-tumor activity of Vitamin D in PDAC. We first examined whether VDR overexpression alone is sufficient to trigger anti-tumor activity of Vitamin D in the Vitamin D-resistant PDAC cells with low VDR expression. Immunoblot analysis showed that the VDR is highly expressed in both cytosol and nucleus in the overexpressed PDAC cells, and CPT treatment increased the nuclear VDR levels (Fig. 6A). Cell proliferation assays demonstrated that VDR overexpression did not trigger anti-proliferative activity of CPT; rather, it slightly increased proliferation capacity (Fig. S11A and Fig. 6B). Meanwhile, cells in which VDR is overexpressed were more sensitive to epigenetic priming compared to unprimed cells (Fig. 6B). Additionally, VDR overexpression significantly increased cell migration in unprimed cells, with or without CPT treatment, but significantly decreased cell migration in the epigenetically primed cells, even in the absence of CPT treatment (Fig. 6C–D and Fig. S11B, C). Intriguingly, VDR overexpression and CPT treatment had comparable effects on decreasing cell proliferation and migration in epigenetically primed cells (Fig. 6B–D), suggesting that a certain level of VDR, either through VDR overexpression or Vitamin D induction, is important for anti-tumor activity under the epigenetic priming conditions. Although VDR overexpression exhibited an obvious tendency to augment the original Vitamin D response in unprimed cells, VDR overexpression did not induce further decreases in cell proliferation and migration in the epigenetically primed cells with CPT treatment (Fig. 6B–D). We speculated that epigenetic priming significantly sensitized the cells to respond to Vitamin D treatment and that the response was saturated. Together, these data suggest that VDR is not sufficient to trigger the anti-tumor activity of Vitamin D, although it is important for augmenting the original Vitamin D response. This observation, in turn, highlights the necessity of Vitamin D-responsive genes in determining the anti-tumor activity of Vitamin D in PDAC, especially when VDR is expressed.

We next sought to determine whether VDR is necessary for the anti-tumor activity of Vitamin D in PDAC cells. CRISPR/Cas9 technology was used to generate a knockout gene of *vdr* in PDAC cells with distinctive Vitamin D responses, including MiaPaca2, Panc1, and BxPC3 cells. Immunoblot showed that genome editing with CRISPR/Cas9 successfully and efficiently eliminated VDR protein expression in these cells (Fig. 6E and Fig. S11D). Although *vdr* knockout did not significantly affect cell viability in unprimed cells with or without CPT treatment, the epigenetically primed MiaPaca2 and Panc1 cells with *vdr* deletion became more resistant to CPT treatment (Fig. 6F). The *vdr* depleted BxPC3 cells also became more resistant to CPT treatment. (Fig. S11E). In addition, *vdr* deletion not only completely blocked the pro-migration effects of CPT in unprimed MiaPaca2 and Panc1 cells but also eliminated the anti-migration effects of CPT upon epigenetic priming (Fig. 6G, H and S11F, G). As for the observation that *vdr* deletion led to decreased migration in the unprimed cells and increased migration in the epigenetically primed cells, we speculated that some undefined level of Vitamin D potentially present in the serum elevated the baseline of cell migration in unprimed cells and decreased it in epigenetically primed cells, which could be blocked by *vdr* deletion. To avoid potential effects from the Vitamin D present in serum and to maximize the beneficial effects of epigenetic priming for enhancing Vitamin D response in Vitamin D-resistant human PDAC cells, as reported by Porter et al. [14], we cultured these cells in a serum-free 3D culture system. Although both MiaPaca2 and Panc1 cells were viable under this culture system, the cells were extremely vulnerable to epigenetic priming with GTA and 5-Aza, so we were unable to investigate the beneficial effects of epigenetic priming on the enhancement of anti-tumor activity by Vitamin D (Fig. S12A, B). Collectively, these data indicate that VDR is necessary for the anti-tumor activity of Vitamin D in PDAC.

## Discussion

One of the key obstacles to PDAC therapy is cell heterogeneity [2, 17]. Transcriptional profiling has characterized PDAC into distinct subtypes, with the majority being the classical or basal-like subtype [43, 44]. Different subtypes of PDAC were originally considered to exist across patients. However, a recent study using dual-color RNA-ISH technology showed that heterogeneity can also be observed in tumor cells from the same patient, as different subtypes of tumor cells expressing different subtype markers exist on a continuum in the same tumor [14]. The findings that dysfunction of some epigenetic regulators induces subtype switching suggests that different subtypes of PDAC are not permanently installed but display a high degree of plasticity [17, 45, 46]. The vast heterogeneity, coupled with strong plasticity, makes it possible for PDAC to be selected or reshaped by drug treatment, leading to widespread therapeutic resistance in patients with PDAC. Indeed, recent evidence shows that Vitamin D, a drug under clinical investigation for PDAC, has subtype-dependent effects on human PDAC cells [14]. Specifically, Vitamin D has favorable effects, such as decreased cell proliferation and migration, in classical subtypes, but is either not effective or causes unfavorable consequences, such as increased cell migration, in basal-like PDAC cells [14]. In addition, VDR expression has also shown an obvious subtype preference in human PDAC cells, such that classical subtypes express higher levels of VDR, whereas basal-like subtypes, such as MiaPaca2 and Panc1, express low levels of VDR [14]. Compelling evidence has shown that the transcriptomic phenotype of PDAC is epigenetically controlled [15, 23]. Epigenetic priming is therefore considered an attractive strategy for targeting tumor heterogeneity and overcoming therapeutic resistance in PDAC. In the present study, we examined the therapeutic potential of epigenetic priming with GTA and 5-Aza, which target histone acetylation and DNA methylation, respectively, to overcome distinctive Vitamin D responses and shift PDAC cells toward a Vitamin D-susceptible state. Intriguingly, our data demonstrate that epigenetic priming not only overcomes Vitamin D resistance on cell proliferation but also shifts the unfavorable effects of Vitamin D from pro-migration toward anti-migration in both MiaPaca2 and Panc1 cells, which are classified as basal-like PDAC cell with low VDR expression [14]. In BxPC3 cells, which are Vitamin D-sensitive PDAC cells with high VDR expression, epigenetic priming also further enhances the anti-proliferative activity of Vitamin D. In addition, epigenetic priming enhances the anti-proliferative activity of Vitamin D in genetically engineered mouse PDAC cells, irrespective of the original Vitamin D responses and VDR expression levels, albeit no obvious subtype-dependent response to Vitamin D has been observed in these cells. Together, these data provide definitive evidence that epigenetic priming with GTA and 5-Aza, at least under in vitro conditions, can be a feasible strategy for targeting tumor heterogeneity and shifting PDAC cells toward a Vitamin D-susceptible state. Moreover, these data further validate that tumor heterogeneity of PDAC is epigenetically controlled.

Epigenetic therapies have shown promising anti-tumor effects in PDAC. For example, combination treatment with HDAC inhibitor SAHA and DNMT inhibitor 5-Aza have resulted in significant delays in tumor growth in vivo by increasing expression of CDKN1A (or P21) [22]. Here, we used GTA, instead of HDAC inhibitors, to increase histone acetylation, especially H3K27ac, by providing substrates for acetyl-CoA. The same effect of increasing CDKN1A was observed in both treatments, suggesting that the silencing of CDKN1A in PDAC is epigenetically controlled [22, 47]. Interestingly, our data also show that TGF-β signaling, which promotes epithelial-mesenchymal transition in PDAC [48–50], is inhibited by GTA and 5-Aza combination treatment, as TGFB2 was significantly downregulated while the TGF-β inactivator NOG was significantly upregulated. Moreover, GSEA analysis showed that epigenetic priming with GTA and 5-Aza upregulates genes that are related to cell death, along with genes involved in negative regulation of cell proliferation and migration. Consistent with the alternations in gene expression, the cell viability and migration capability of PDAC were significantly inhibited by GTA and 5-Aza combination treatment. Together, these results provide further evidence for the concept that epigenetic treatment is a promising strategy for combating PDAC. Moreover, it is worth mentioning that the anti-tumor activity of epigenetic priming with GTA and 5-Aza may partially rely on the Vitamin D signaling, as *vdr* deletion led to enhanced cell migration in the epigenetically primed PDAC cells (Fig. 6G, H).

The first attempts to elucidate the anti-tumor activity of Vitamin D in PDAC dates to the 1990s, when distinctive responses to Vitamin D treatment were observed in different PDAC cell lines [10–13]. However, until recently, the underlying mechanisms have puzzled researchers in the field for about 30 years. Owing to developments in next-generation sequencing technology, we can now stratify PDAC heterogeneity into distinct subtypes [43, 44] and demonstrate that both VDR expression and Vitamin D responses are subtype-dependent in human PDAC [14]. As the phenotypic heterogeneities between subtypes are hierarchically installed and controlled by epigenetic cues, we hypothesize that the distinctive responses to Vitamin D treatment between subtypes are determined by the epigenetic state of the tumor cells. Indeed, epigenetic reprograming with GTA and 5-Aza eliminates the heterogeneities of Vitamin D response in PDAC, as consistently enhanced anti-tumor activity of Vitamin D is observed in epigenetically primed PDAC cells, regardless of the original Vitamin D response or VDR expression. Transcriptional analysis reveals that increasing global H3K27ac with GTA and decreasing global DNA methylation with 5-Aza not only significantly increase VDR expression but also comprehensively reprogram Vitamin D-responsive genes. Vitamin D induced phenotypic alternations are tightly associated with reprogrammed Vitamin D-responsive genes. In addition, gain-of-function experiments shows that VDR overexpression only augments the phenotypic characteristics in PDAC cells but does not appreciably shift the phenotypic characteristic to another, suggesting that VDR is not responsible for triggering the anti-tumor activity of Vitamin D. In turn, these data highlight that the Vitamin D-responsive genes play a key role in the anti-tumor activity of Vitamin D, especially when the VDR is available. This finding is consistent with the observations that VDR expression levels are not closely correlated with the Vitamin D response in genetically engineered mouse PDAC cells. Moreover, loss-of-function experiments validate the observation that VDR is necessary for Vitamin D response in PDAC. Therefore, we propose a previously undefined mechanism that both the VDR and Vitamin D-responsive genes are prerequisites for Vitamin D response, and the epigenetic states of both the VDR and Vitamin D-responsive genes play central roles in orchestrating the expression of these genes and determining the anti-tumor activity of Vitamin D in PDAC.

In summary, our study demonstrated that epigenetic treatment with GTA and 5-Aza is a feasible strategy for overcoming PDAC heterogeneity in Vitamin D response, and for shifting PDAC cells toward a Vitamin D-susceptible state. This finding expands the evidence that PDAC heterogeneity is epigenetically controlled, and that epigenetic therapy is a promising strategy for PDAC treatment. Importantly, but not unexpectedly, we were able to show that both the VDR and Vitamin D-responsive genes are the prerequisites for Vitamin D response. The epigenetic state that controls the expression of both the VDR and Vitamin D-responsive genes is the determining factor for the anti-tumor activity of Vitamin D in PDAC.

## Limitations of the study

As we did not exclude the effects of the Vitamin D in the serum used for cell culture, the beneficial effects of epigenetic priming on the therapeutic effects of Vitamin D may be underestimated.

To investigate the possible use of epigenetic reprogramming to overcome the inconsistency of Vitamin D responses between PDAC subtypes, we utilized GTA, but not the more toxic HDAC inhibitors, and 5-Aza to shift the transcriptomic phenotype of PDAC toward a Vitamin D-susceptible state. Although this strategy enabled us to observe the enhanced anti-tumor activity of Vitamin D in PDAC cells by epigenetic priming, extension to an in vivo system is complicated due to several challenges. For example, the strict concentration demands of both GTA and 5-Aza for observing the Vitamin D effects in vitro means that it would be difficult to validate this strategy in vivo, due to the challenges of controlling the amount of drugs delivered to the tumor in vivo. The effectiveness of 5-Aza in treating solid tumors has been less than optimal. Therefore, we plan to assess niclosamide ethanolamine (NEN), a mitochondrial uncoupler that has recently been shown to remodel cancer epigenome [51], for its potential to induce DNA demethylation in PDAC models. In addition, we and others [27, 52] have shown that GTA has a short metabolic half-life (less than 2 h) in the blood of mice, which may create an additional obstacle for in vivo experiments.

## Materials and methods

### Cell culture and drug treatment

All the cell lines, including primary MEFs, human and mouse PDAC cell lines, were obtained from Dr. Laura Attardi’s laboratory (Stanford University). The primary MEF cells were generated from E13.5 mouse embryo of 129/SvJ strain. The primary MEF cells were used at the early passages (3–5 passages). The human PDAC cell lines were originally from ATCC. The four Mouse PDAC cell lines were generated from the PDAC tissues grown in mixed background mice with the indicated genotypes [42]. The information of the strains are as follows: Sox9CreER (RRID:IMSR_JAX:018829), Ptf1aCreER(RRID:IMSR_JAX:019378), KrasLSL-12D (RRID:IMSR_JAX:008179), Trp53fl (RRID:IMSR_JAX:008462), Trp53LSL-R172H (RRID:IMSR_JAX:008652) and Rosa26LSL-tdTomato (RRID:IMSR_JAX:007909). All Cell lines were tested negative for Mycoplasma (MycoAlert Mycoplasma Detection Kit; Lonza). All cells were maintained at 37 °C under a humidified atmosphere containing 5% CO_2_ and cultured in DMEM/F12 (Gibco; 2508902) with 10% fetal bovine serum (FBS) (Sigma; 21C815), 1% penicillin-streptomycin (Gibico; 15140122) and extra 2.5mM l-Glutamine (Gibico; 25030081). All Cell lines used for the experiment were passaged no more than 10 times from the time of thawing. For drug treatments, calcipotriol (sc-203537; 10 mg), EB1089 (sc-358831; 1 mg), glyceryl triacetate (90240; 250 ml) and 5-Aza-2-′deoxycytidine (A3656; 5 mg) were obtained from Sigma-Aldrich.

### Cell proliferation and viability assay

Cell proliferation was assessed using a hemocytometer. A total of 5 × 10^4^ human PDAC cells and 3 × 10^4^ genetically engineered mouse PDAC cells were plated in 12-well plate and allowed to attach overnight. The media were subsequently replaced with fresh media containing either DMSO or indicated drugs. After 72 h of treatment, the media from each well was collected and the attached cells were dissociated with 200 µl Trypsin (Thermo Scientific). Each well of dissociated cells was combined with its corresponding collected media, spun down with 2000 × *g* for 5 min at 4 °C, after which the supernatant was removed and the cells were re-suspended in cell culture media containing 10% FBS, to which was added the same volume of 0.4% Trypan Blue Stain (Thermo Scientific) to distinguish live from dead cells. Next, 10 µl cell suspensions were loaded into the hemocytometer, and the cells were counted under a light microscope. As previous reported [28, 53], the proliferation assay for primary MEF cells and 293 T cells were automatically determined using Agilent Cytation5. 9.0 × 10^4^ primary MEF cells and 3.0 × 10^4^ 293 T cells were plated in 24-well plates (Corning) and attached overnight. Then, the fresh media containing either DMSO or indicated drugs were used to replace the old media. After 72 h treatment, cells were stained with 20 μM Hoescht 33342 reagent for 10 min prior to counting with Cytation5.

### Protein isolation and immunoblot analysis

Cells were washed and collected using cold PBS buffer. To isolate cytosolic proteins, cells were lysed with Harvest lysis buffer (10 mM HEPES pH = 7.9, 50 mM NaCl, 500 mM sucrose, 0.1 mM EDTA, 0.5% Triton x 100) for 10 min over ice, and the cell lysates were spun down at 2000 × *g* for 5 min at 4 °C and the supernatants containing the cytosolic proteins to a new tube. The insoluble pellets containing nuclear proteins were further lysed with nuclear lysis buffer (10 mM HEPES pH = 7.9, 500 mM NaCl, 0.1 mM EDTA, 0.1 mM EGTA, 0.1% NP40) for 10 min over ice. The lysates were sonicated with a Biorupter Plus sonication device (Diagenode) for 15 cycles (1 cycle = 60 s on and 60 s off), and the sonicated nuclear lysates were spun down at 20,000 × *g* for 10 min at 4 °C. The supernatants containing nuclear proteins were subsequently transferred to a new tube. To obtain the whole cell proteins, cells were lysed with strong RIPA buffer (50 mM Tris-HCl pH 7.5, 300 mM NaCl, 1% SDS, 1% NP40, 1 mM EDTA) for 10 min over ice, the lysates were sonicated for 15 cycles, and the sonicated cell lysates were spun down with 20,000 × *g* for 10 min at 4 °C. The supernatants containing both cytosolic and nuclear proteins were subsequently transferred to a new tube. All lysis buffers were freshly supplemented with Halt Protease and Phosphatase Inhibitor Cocktail (Thermo Scientific) and 1 mM PMSF (Sigma) before use. Protein concentration was determined by BCA assay according to the manufacturer’s instruction. Equal amounts of protein were denatured with loading buffer at 98 °C and then separated by 4–12% Bis-Tris Protein Gels (Thermo Scientific). The proteins were then transferred to nitrocellulose membranes (Thermo Scientific). The membranes with protein blots were blocked with 5% non-fat milk for 1 h and then incubated with the first antibody overnight at 4 °C. The membrane was washed with TBST and incubated with HRP-conjugated secondary antibodies for 1 h at room temperature. Signals were detected with an ECL kit. All the antibodies were diluted in TBST that was supplemented with 3% bovine serum albumin (Equitech-Bio). Anti-VDR antibody (Cell Signaling Technology; 12550s) was diluted at a ratio of 1:1000. Anti-H3K27ac antibody (Active Motif; 39133), anti-H3K4ac antibody (Millipore; 07-539), anti-H3K9ac antibody (Cell Signaling Technology; 9649s), anti-H3Kac antibody (Millipore; 06–599) and anti-total Kac (Cell Signaling Technology; 9681s) were diluted at a ratio of 1:2000. Anti-β-actin antibody (Cell Signaling Technology; 3700s) and anti-H3 antibody (Cell Signaling Technology; 3638s) were diluted at a ratio of 1:10000.

### Measurement of intracellular acetyl-CoA with LC–MS

Cells were washed with cold PBS in the dish three times. Added 500 µl 80% LC–MS grade methanol (Thermo Scientific) to collect the cells and sonicated the cells with Biorupter Plus sonication device (Diagenode) for 2 cycles (1 cycle = 30 s on and 30 s off), the sonicated cell-methanol mixture was kept for 1 h at −20 °C and then centrifuged with 20,000 × *g* for 10 min at 4 °C. Supernatants were collected for liquid chromatography-mass spectrometry (LC–MS) analysis and the pellet was subjected to protein concentration analysis with the BCA assay. The LC–MS analysis was conducted as previously described [27]. Liquid chromatography was performed with an Agilent 1290 Infinity LC system (Agilent, Santa Clara, US) coupled to a Q-TOF 6545 mass spectrometer (Agilent, Santa Clara, US). A hydrophilic interaction chromatography method with a ZIC-pHILIC column (150 × 2.1 mm, 5 μm; EMD Millipore) was used for compound separation at 25 °C with a flow rate of 0.3 mL/min. Mobile phase A was 20 mM ammonium carbonate in LC-MS grade water and mobile phase B was 100% acetonitrile. The gradient elution was 0–1.5 min, 80% B; 1.5–7 min, 80% B → 50% B, 7–8.5 min, 50% B; 8.5–9.0 min, 50% B → 80% B. After the gradient, the column was re-equilibrated at 80%B for 11 min. The overall runtime was 20 min, and the injection volume was 5 μL. The Agilent Q-TOF was operated in negative mode and the relevant parameters were as listed: ion spray voltage, 3500 V; nozzle voltage, 1000 V; fragmentor voltage, 125 V; drying gas flow, 11 L/min; capillary temperature, 325 °C; drying gas temperature, 350 °C; and nebulizer pressure, 40 psi. A full scan range was set at 50–1600 (m/z). The reference masses were 119.0363 and 980.0164. The acquisition rate was 2 spectra/s. Targeted analysis was performed to extract the abundance of metabolites with Agilent Profinder B.10.00 software (Agilent Technologies). The abundance of acetyl-CoA was normalized according to protein level.

### RNA isolation, reverse transcription, and quantitative real-time PCR

Total RNA was isolated from the cells using the TRIzol Reagent (Invitrogen) protocol. One microgram of total RNA was used to synthesize cDNA using the SuperScript™ First-Strand Synthesis System for RT-PCR (Invitrogen). Expression levels of the genes were measured at the Prism 7900 Sequence Detection System (Applied Biosystems) using SYBR green qPCR master mix (Thermo Scientific). The mRNA levels were normalized to 18S rRNA. The primer information for the qRT-PCR experiment was the following: VDR Forward: TCCAGTTCGTGTGAATGA, Reverse: AGGGTCATCTGAATCTTCTT; 18S rRNA Forward: GAGGATGAGGTGGAACGTGT, Reverse: AGAAGTGACGCAGCCCTCTA.

### DNA preparation, dot blot assay and MeDIP-qPCR assay

Cells were treated with the indicated drugs for 24 h. The cells were subsequently digested with lysis buffer (50 mM Tris-HCl pH 8.0, 1% SDS, 1 mM EDTA) containing 20 mg/ml proteinase K at 55 °C for overnight. One volume of phenol, chloroform, and isogamy alcohol (in a ratio of 25:24:1) were added to the samples. The samples were vortexed thoroughly and centrifuged at room temperature for 5 min at 20,000 × *g*. The upper aqueous phase was transferred to a fresh tube. One volume of isopropanol was added to precipitate the DNA, and the isolated DNA pellets were subsequently washed with 70% ethanol. The DNA were dried and re-dissolved in TE buffer (10 mM Tris-HCl pH 8.0, 1 mM EDTA).

For dot blot assay, the genomic DNA was diluted with 20× SSC buffer and denatured for 10 min at 95 °C. Samples were rapidly chilled on ice and then spotted on a positive charged nylon membrane. The membrane was washed twice with SSC buffer, subjected to UV crosslink and dried for 1 h at 70 °C. The dried membrane containing DNA was blocked with 5% milk and then incubated with anti-5mC antibody (Diagenode, C15200081, dilution at 1:2000) at 4 °C overnight. The following procedures are same as those for the immunoblot analysis. The dried membrane containing DNA stained with methylene blue staining buffer (0.2% methylene blue in 0.4 M sodium acetate and 0.4 M acetic acid) was used for evaluating the DNA loading.

For MeDIP-qPCR assay, the genomic DNA was sonicated to random fragments of 200–500 bp with a Biorupter Plus sonication device (Diagenode) for 20 cycles (1 cycle = 10 s on and 50 s off). Take 1% sonicated DNA fragments (10 µl) as input (normalized control), the remaining sonicated DNA fragments were divided in two (1 ml for each), one part was incubated with mouse IgG antibody (Diagenode; C15400001-100) and the other was incubated with anti-5mC antibody (Epigentek; A-1014-100). MeDIP was performed using protein G Dynabeads (Thermo Scientific). The immunoprecipitated Beads-DNA was sequencing washed with Low Salt Wash Buffer (0.1% SDS, 1% Triton X-100, 2 mM EDTA, 20 mM Tris-HCl, pH 8.0, 150 mM NaCl), High Salt Wash Buffer (0.1% SDS, 1% Triton X-100, 2 mM EDTA, 20 mM Tris-HCl, pH 8.0, 50 mM NaCl), LiCl Wash Buffer (0.25 M LiCl, 1% NP40, 1% sodium deoxycholate, 1 mM EDTA, 10 mM Tris-HCl, pH 8.0) and TSE Buffer (10 mM Tris-HCl, pH 8.0, 1 mM EDTA, 50 mM NaCl), and eluted with elution buffer (1% SDS, 50 mM Tris-HCl, pH 8.0, and 10 mM EDTA). The eluted DNA and input DNA were purified by the above-mentioned phenol chloroform extraction method. The qRT-PCR method was same with mRNA qPCR experiments. The input percentage for each MeDIP fraction was calculated using the following formula:

$${Input} \% =100\times {2}^{-({CtMeDIP}-({Ctinput}-\log 2({Dilution}{factor})))}$$

As the input DNA is 1%, the dilution factor here is 100.

The primer information for the qRT-PCR is as following: VDR L1 site Forward: CTCGCCAACCTGTTACTG, Reverse: CTCCCTTGGGTGAGATTC; CDKN1A L1 site Forward: GTAGGGTGTAGGGAGATTG, Reverse: ATTGAGGTCCACTGAACTTA; DUSP10 L1 site Forward: CTCCTTACTATGGACAGATGA, Reverse: CGTTTGTCAAGTCACCTC; DUSP10 L2 site Forward: AGCATCGCATCTCTATCTT, Reverse: GGAGGTGGAGGATTGTAA; MT1G L1 site Forward: GCCTACCCCAGTATTCTG, Reverse: GAAAGTGGAGCACAAGAC; MT1G L2 site Forward: CTCTATGGTGTCTGGGAAT, Reverse: GAACTCTAGTCTCGCCTC.

### RNA sequencing and data analysis

Cells were treated with the indicated drugs for 24 h. Total RNA was subsequently isolated from cells using TRIzol Reagent (Invitrogen) protocol (*n* = 3 per group). The RNA-seq library was constructed and subjected to 150 base-pair paired-end sequencing on an Illumina sequencing platform from Novogene. Row data were trimmed and filtered with trim galore (https://github.com/FelixKrueger/TrimGalore). High quality and clean data were aligned to the human reference genome GRCh38 using hisat2 (version: 2.2.1) [54]. HTseq (version: v2.0.1) [55] and StringTie (version: v2.1.5) [56] were used to calculate the read counts and the TPM value, respectively.

As described previously [57], the EdgeR R library was used for differential expression analysis [58]. Differential expression genes were identified as having a fold change significantly greater than 1.5 at *P* value < 0.05. Volcano Plots and Venn Diagrams were drawn by the ggplot2 R library. Gene ontology (GO) enrichment analysis of the DEGs was carried out by DAVID (the Database for Annotation, Visualization, and Integrated Discovery, https://david.ncifcrf.gov) [59]. Gene Set Enrichment Analysis (GSEA) was performed to determine the global gene expression tendency for a priori defined set of genes [60]. All the gene sets were from the GO database with the indicated GO ID.

### ChIP-qPCR analysis

As previously reported [28], MiaPaca2 cells (2 × 10^7^) were fixed for 10 min in 1% formaldehyde followed by reaction quenching with 125 mM glycine for 10 min. Cells were lysed in lysis buffer (10 mM Tris–HCl PH = 8.0, 100 mM NaCl, 1 mM EDTA, 0.5 mM EGTA, 0.1% sodium deoxycholate, and 0.5% N-laurylsarcosine) supplemented with 1 mM PMSF (Sigma) and 1:100 Halt Protease and phosphatase Inhibitor Cocktail (Thermo Scientific). The DNA was sonicated to random fragments of 200–800 bp with a Q500 Sonicator (Qsonica) using a 2 mm probe at 20% amplitude for 40 cycles (1 cycle = 5 s on and 45 sec off). Take 1% sonicated DNA fragments (10 µl) as input. The remaining sonicated DNA fragments were divided in two (1 ml for each) and then diluted with 4 ml dilution buffer (10 mM Tris-HCl (PH = 8.0), 100 mM NaCl, 1 mM EDTA, 0.5 mM EGTA, 0.1% sodium deoxycholate, and 0.125% Triton-x 100)). One part was incubated with rabbit IgG antibody (Diagenode; C15410206) and the other was incubated with anti-H3K27ac antibody (Active Motif; 39133). DNA was pulled down by using protein G Dynabeads (Thermo Scientific). The immunoprecipitated Beads-DNA then washed and eluted using the same method with MeDIP. To reverse cross-links, DNA were incubated at 65 °C overnight followed by incubation with Rnase A (Thermo Scientific) at 37 °C for 30 min and 20 mg/ml proteinase K (Thermo Scientific) at 55 °C for 2 h. DNA extraction and qPCR experiments were conducted same with MeDIP-qPCR experiments. The primer information for the qRT-PCR is as following: VDR Forward: CTCTGGGACTTTGGGATAT, Reverse: TGTATCTTCAGCGAGGTG; CDKN1A Forward: GAACGGACTGTATGAGGT, Reverse: AGCCAAATAGGTCACTGT; DUSP10 Forward: AGCATCGCATCTCTATCTT, Reverse: GGAGGTGGAGGATTGTAA; CNN3 Forward: CTCCAGGAAAACGGTGAG, Reverse: CGAAGTCAAGAACAAGGTAC; CXCL3 Forward: CTCTGGGACTTTGGGATAT, Reverse: TGTATCTTCAGCGAGGTG.

### Molecular cloning, virus production and infection

VDR cDNA was subcloned to pCDH-CMV vector (Addgene) using standard molecular biology techniques. Guide RNA (GAACGTGCCCCGGATCTGTG) against human VDR was cloned into pLentiCRISPRv2 vector (Addgene). As previously reported, the guide RNA (CACCGGCACTACCAGAGCTAACTCA) was used as a control. The psPAX2 and pMD2.G packaging plasmids (Addgene) were used to produce Lentiviral particles. 293T cells were transfected with the indicated plasmids using Lipofectamine 3000 transfection reagent (Thermo Scientific). After a change of medium at 8 h post transfection (hpt), the supernatant containing the viruses was harvested on 56 hpt and 80 hpt. The viruses were aliquoted and stored at −80 °C after filtering with 0.45 µM filters. Target cells were infected with the viruses in the presence of 8 µg/ml polybrene. Puromycin was used to select the infected cells.

### Migration assays

Cells were treated with the indicated drugs for 24 h and dissociated to single-cell suspensions with Trypsin (Thermo Scientific). The dissociated cells were collected by centrifuge and resuspended with serum-free media. Cells in serum free media were seeded onto 8-µM pore Transwell membrane (Corning) at 70000 MiaPaca2 cells per well or 100000 Panc1 cells per well in a 24-well plate containing complete growth media (with 10% FBS) in the bottom chamber. After 15 h of culture at 37 °C under a humidified atmosphere containing 5% CO_2_, unmigrated cells on top of the chamber were stripped off with cotton swabs, and cells on the bottom of the chamber were fixed with 80% methanol and stained with DAPI. Images were acquired with Leica fluorescent microscope, and total DAPI positive cells in the image areas were counted using counting tools in Adobe Photoshop. For the cell viability analysis in the migration assays, pretreated cells were dissociated and collected as mentioned above, 1 × 10^6^ cells in serum free medium were seeded into 12-well plates. After 15 h of culture, cells were collected and stained by Trypan Blue stain. As mentioned above, Live cells and dead cells were counted using the hemocytometer method. The cell migration capacity was normalized according to survival using the following formula:

$${{\rm{Migration}}}_{{\rm{Norm}}}={{\rm{Migration}}}_{{\rm{Count}}}\div\frac{{\rm{Live}}}{{\rm{Live}}+{\rm{Dead}}}$$

“Migration_Count_” is the number of cells that pass the transwell membrane; “Live” is the number of live cells that could not be stained by Trypan Blue; “Dead” is the number of dead cells that were stained blue by Trypan Blue.

### Statistical analysis

Unless otherwise specified, each treatment group consists of three replicates. All data are presented as Mean ± SEM. Each experiment included three independent samples. *P* values were calculated using unpaired t test, or one-way, or two-way ANOVA analysis in GraphPad PRISM8 (n.s. no significance or *P* > 0.05; *P < 0.05; **P < 0.01; ***P < 0.005; ****P < 0.001).

### Supplementary information

Supplementary Figures and legends

original data

Checklist

## Data Availability

The Gene Expression Omnibus (GEO) accession no. for the RNA-Seq data produced in this study is GSE247389. The full and uncropped western blots data in this study is available in the supplemental material.
