# Supplementary material for: Epigenetic priming targets tumor heterogeneity to shift transcriptomic phenotype of pancreatic ductal adenocarcinoma towards a Vitamin D susceptible state
Source: Cell Death Dis. 2024 Jan 26;15(1):89. doi: 10.1038/s41419-024-06460-9 (PMC10810848; doi:10.1038/s41419-024-06460-9)
Supplement: Supplementary file 1 — Supplementary Figures and legends [file 41419_2024_6460_MOESM1_ESM.docx]

**
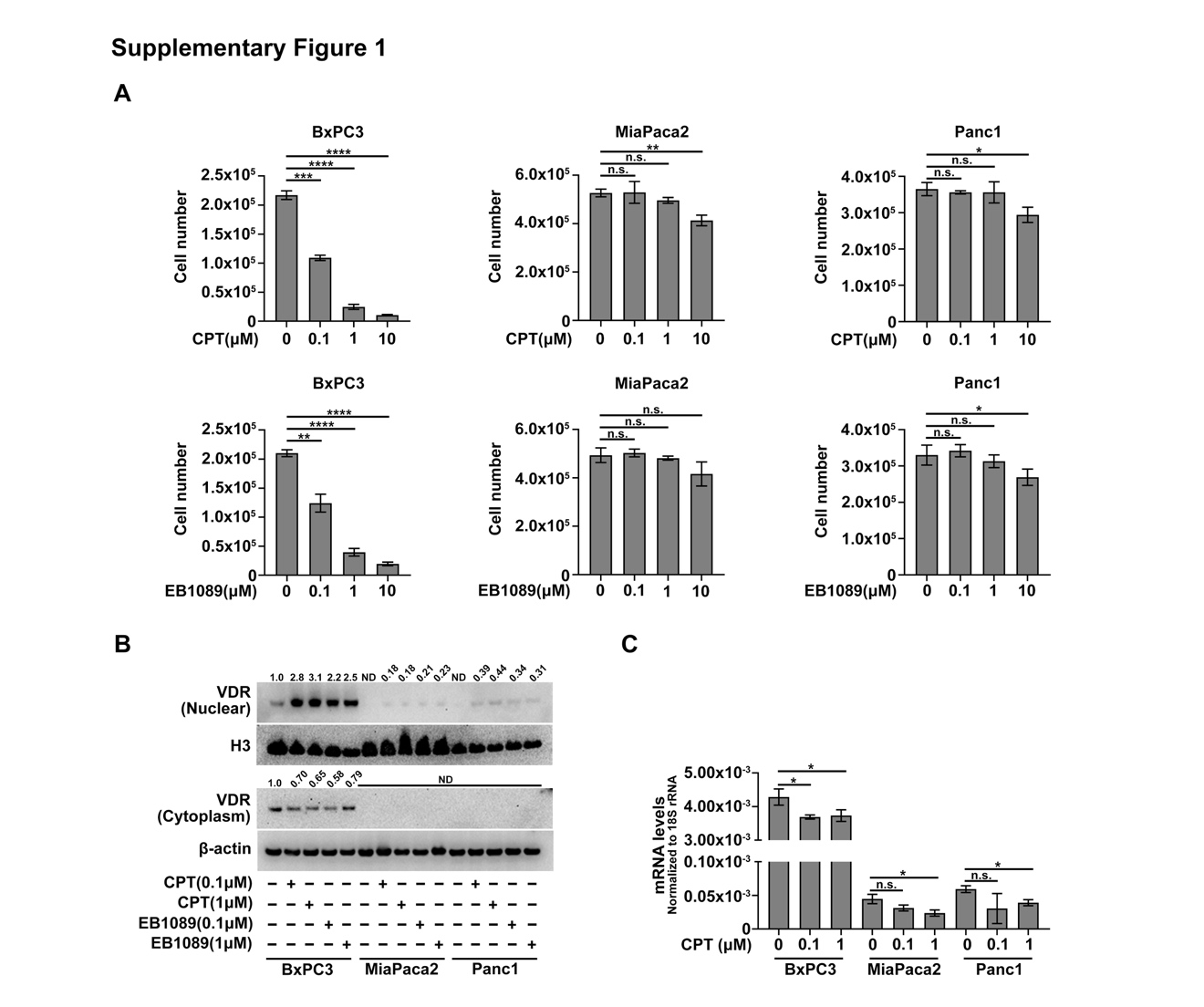
**

**Supplementary Fig. 1. Human PDAC cell lines exhibit distinctive Vitamin D responses and VDR expression.** (A) Human PDAC cells were treated with a series of dilutions of Vitamin D analog CPT (Upper) and EB1089 (Bottom), and cell numbers were counted after 72 h treatment. (B) Immunoblot analysis of cytosolic and nuclear VDR levels in human PDAC cells with indicated treatment. (C) Quantitative real-time PCR analysis in the human PDAC cells treated with different concentrations of CPT; mRNA levels were normalized to 18S rRNA. ND: not detected or not determined. All data are plotted as Mean ± S.E.M. Statistical significance was determined by one-way ANOVA with Tukey’s multiple comparisons test. n.s. no significance or P > 0.05; *P < 0.05; **P < 0.01; ***P < 0.005 and ****P < 0.001.


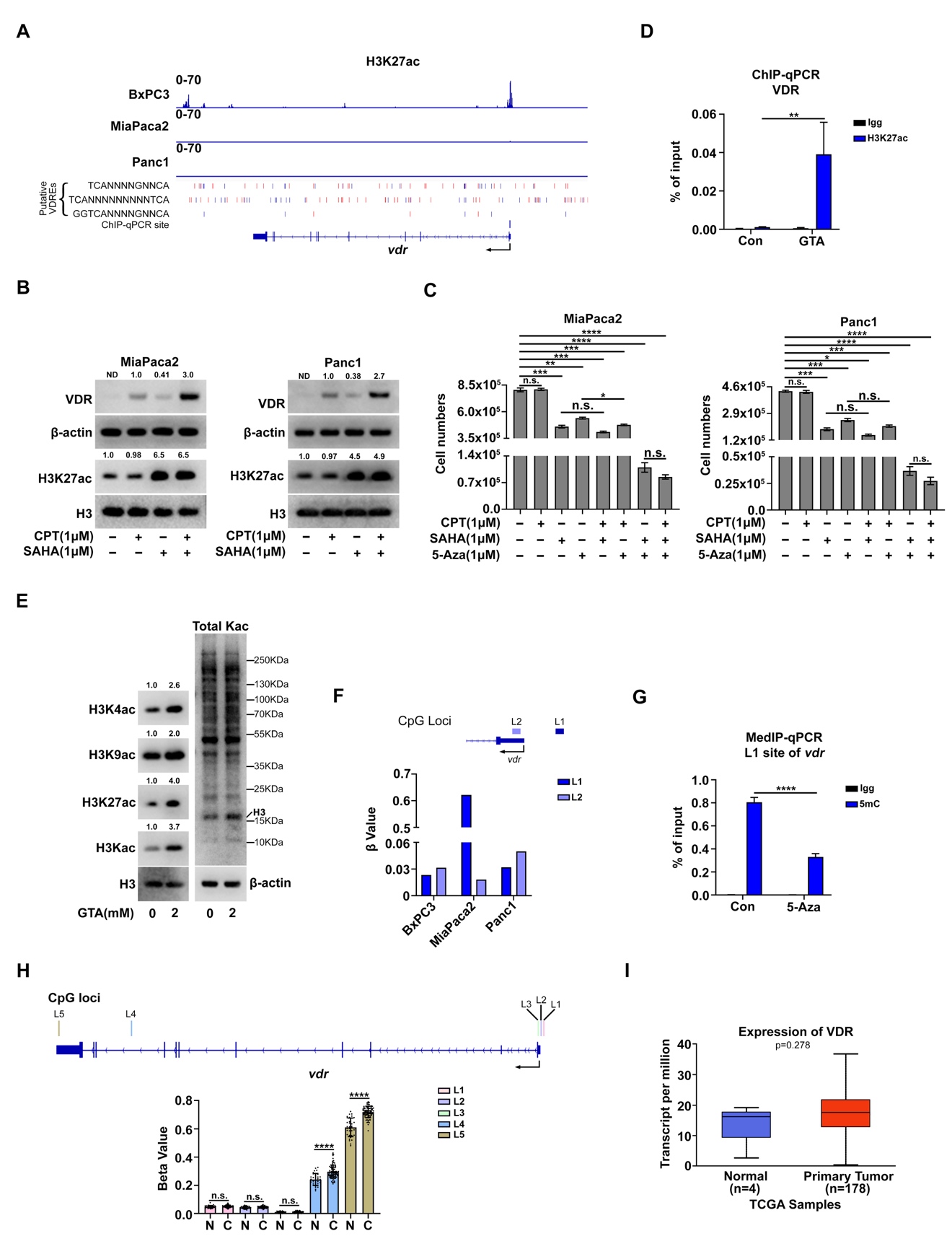


**Supplementary Fig. 2. The VDR is silenced through H3K27 hypo-acetylation and DNA hyper-methylation in MiaPaca2 and Panc1 cells.** (A) ChIP-Seq tracks for H3K27ac surrounding *vdr* (chr12:48,221,084-48,301,753) in the indicated human PDAC cells, raw data was obtained from GEO dataset GSE115463, VDRE: Vitamin D receptor responsive element (61, 62). (B) Immunoblot analysis of whole cell lysates from MiaPaca2 (left) and Panc1 (right) cells with indicated treatment. ND: not detected or not determined. (C) 72-hour proliferation assay of MiaPaca2 and Panc1 cells with the indicated treatment. (D) ChIP-qPCR for H3K27ac at the promoter region of *vdr* gene, the ChIP-qPCR site is indicated in (A). (E) Immunoblot analysis of whole cell lysates from MiaPaca2 cells with indicated treatment. (F) The β values surrounding the *vdr* gene, data was obtained from GEO dataset GSE40099. (G) MeDIP-qPCR for 5mC at the L1 site of the *vdr* gene. The L1 site is indicated in (F). (H) DNA methylation level of CpG loci in *vdr* gene from clinical samples; N indicates non-cancerous normal pancreas tissue and C indicates cancer tissue from PDAC patient; Data was obtained from GEO dataset GSE155353, for normal tissue, n=34, for cancerous tissue, n=82. (I) Expression level of VDR in normal tissue and primary tumor tissue, data was obtained from UALCAN database. All data are plotted as Mean ± S.E.M. Statistical significance was determined by one-way ANOVA with Tukey’s multiple comparisons test for (C) and two-way ANOVA with Sidak’s multiple comparisons test for (D) and (G). n.s. no significance or P > 0.05; *P < 0.05; **P < 0.01; ***P < 0.005 and ****P < 0.001.


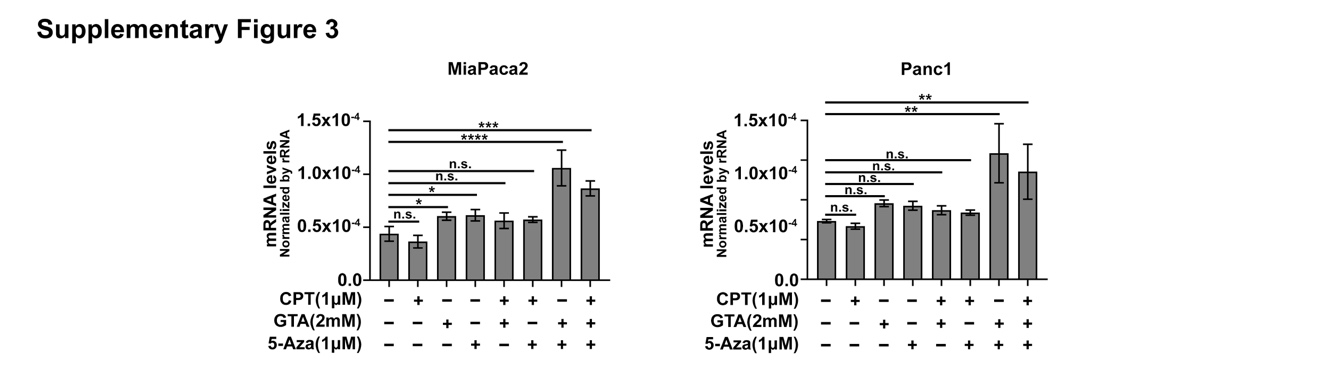


**Supplementary Fig. 3. Epigenetic priming with GTA and 5-Aza elevates the mRNA level of *vdr*.** All data are plotted as Mean ± S.E.M. Statistical significance was determined by one-way ANOVA with Tukey’s multiple comparisons test. n.s. no significance or P > 0.05; *P < 0.05; **P < 0.01; ***P < 0.005 and ****P < 0.001.


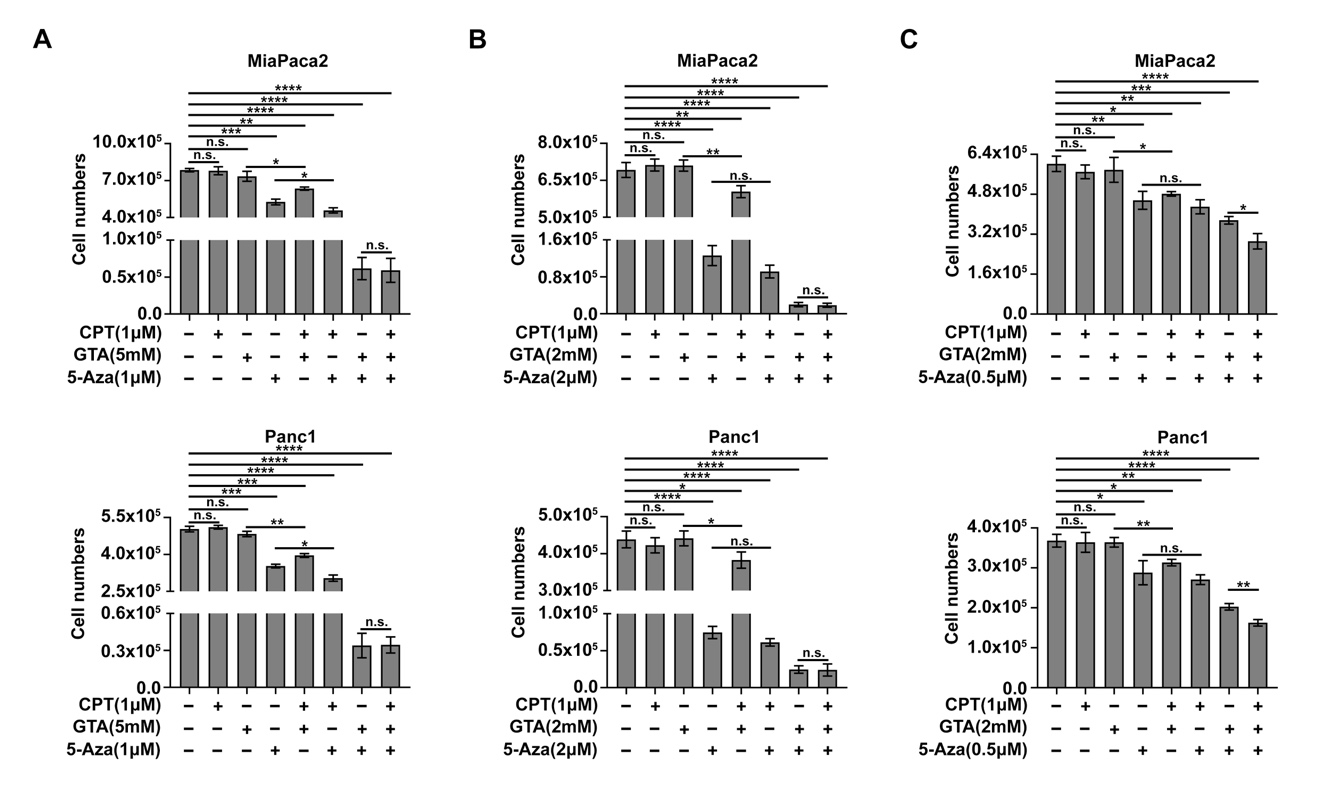


**Supplementary Fig. 4. 72-hour-proliferation assay of human PDAC cells with the indicated treatment.** Neither higher concentration of GTA (A) and 5-Aza (B) nor lower concentration of 5-Aza (C) could further enhance the anti-proliferative activity of CPT. All data are plotted as Mean ± S.E.M. Statistical significance was determined by one-way ANOVA with Tukey’s multiple comparisons test. n.s. no significance or P > 0.05; *P < 0.05; **P < 0.01; ***P < 0.005 and ****P < 0.001.


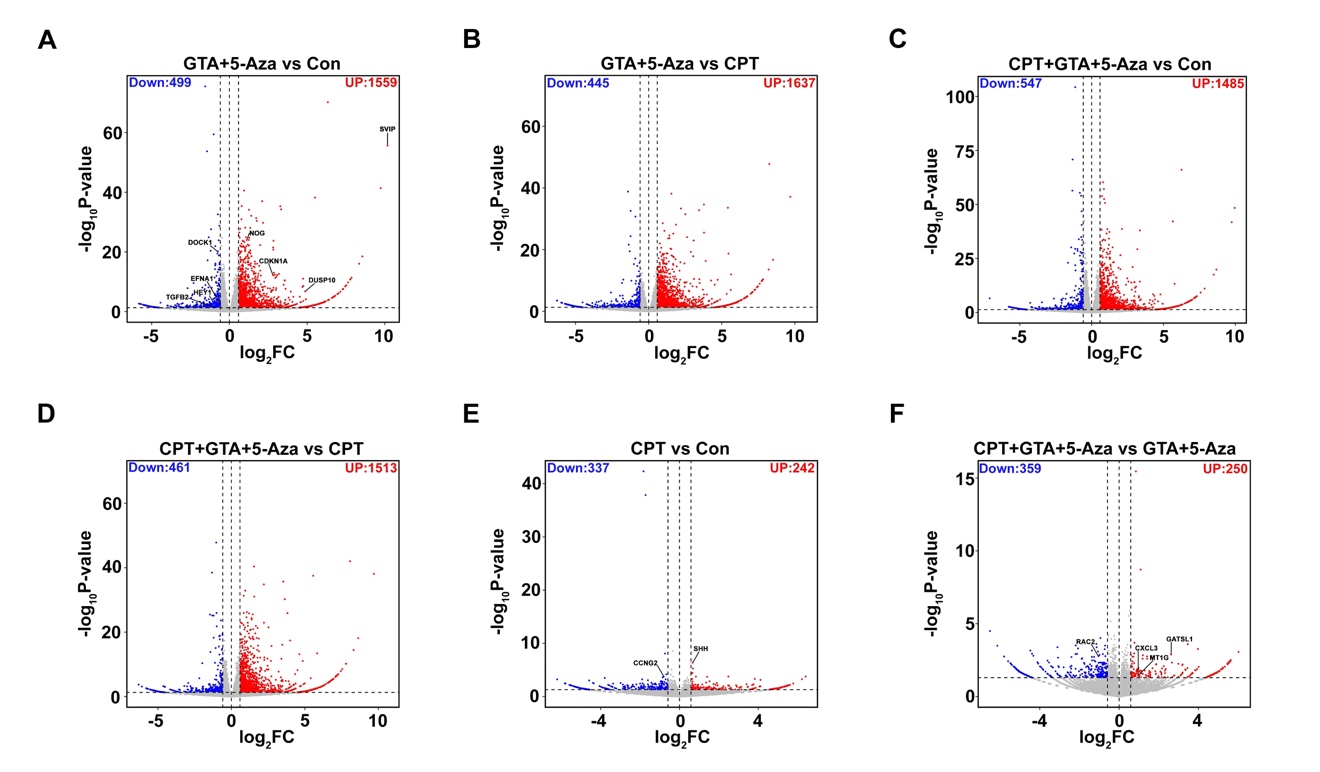


**Supplementary Fig. 5. Volcano plot of the differentially expressed genes (DEGs).** (A) GTA+5-Aza vs. Con; (B) GTA+5-Aza vs. CPT; (C) CPT+GTA+5-Aza vs. Con; (D) CPT+GTA+5-Aza vs. CPT; (E) CPT vs. Con; (F) CPT+GTA+5Aza vs. GTA+5-Aza. Numerals indicate the number of genes up-regulated (red) or down-regulated (blue). The concentration of CPT, GTA, and 5-Aza used in the RNA-Seq experiment was 1µM, 2mM and 1 µM, respectively. All cells received the indicated treatment for 24 h.


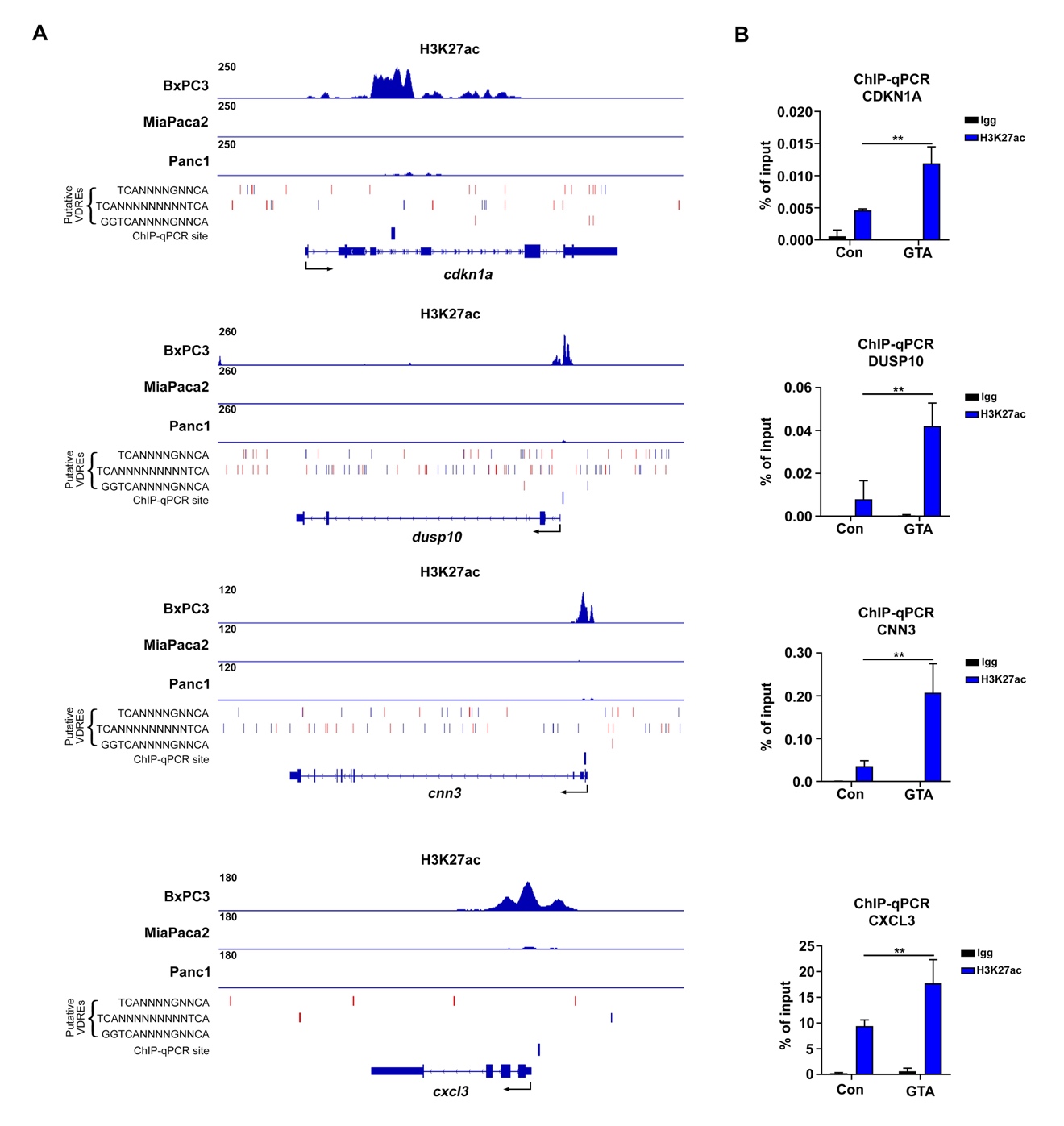


**Supplementary Fig. 6. The representative upregulated genes by epigenetic priming are characterized with H3K27 hypo-acetylation in MiaPaca2 cells.** (A) ChIP-Seq tracks for H3K27ac of representative upregulated genes in the indicated human PDAC cells, data was obtained from the same source as Fig S2A. (B) ChIP-qPCR for H3K27ac at the promoter region of the indicated upregulated genes, the ChIP-qPCR sites are indicated in (A). All data are plotted as Mean ± S.E.M. Statistical significance was determined by two-way ANOVA with Sidak’s multiple comparisons test. **P < 0.01 and ****P < 0.001.


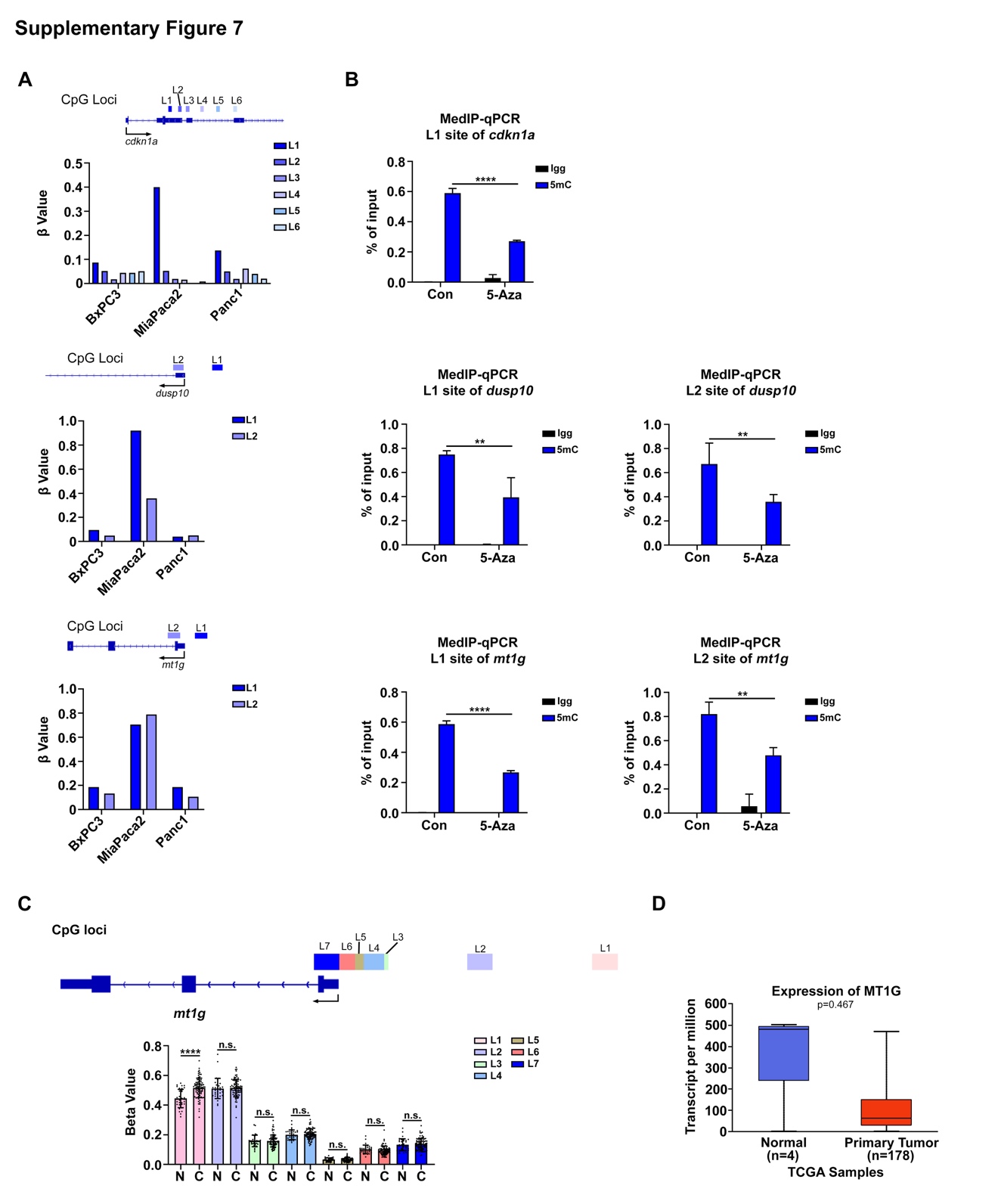


**Supplementary Fig. 7. The representative upregulated genes by epigenetic priming are characterized with DNA hyper-methylation in MiaPaca2 cells.** (A) The β values of CpG loci in the indicated upregulated genes, data was obtained from the same source as Fig S2F. (B) MeDIP-qPCR for 5mC at the indicated CpG loci of the upregulated genes. The exact CpG loci are shown in (A). (C) DNA methylation level of CpG loci in *mt1g* gene from clinical samples, data was obtained from the same source as Fig S2H. (D) Expression level of MT1G in normal tissue and primary tumor tissue, data was obtained from the same source as Fig S2I. All data are plotted as Mean ± S.E.M. Statistical significance was determined by two-way ANOVA with Sidak’s multiple comparisons test for (B) and unpaired t-test for (C and E). n.s. no significance or P > 0.05; **P < 0.01; ***P < 0.005 and ****P < 0.001.


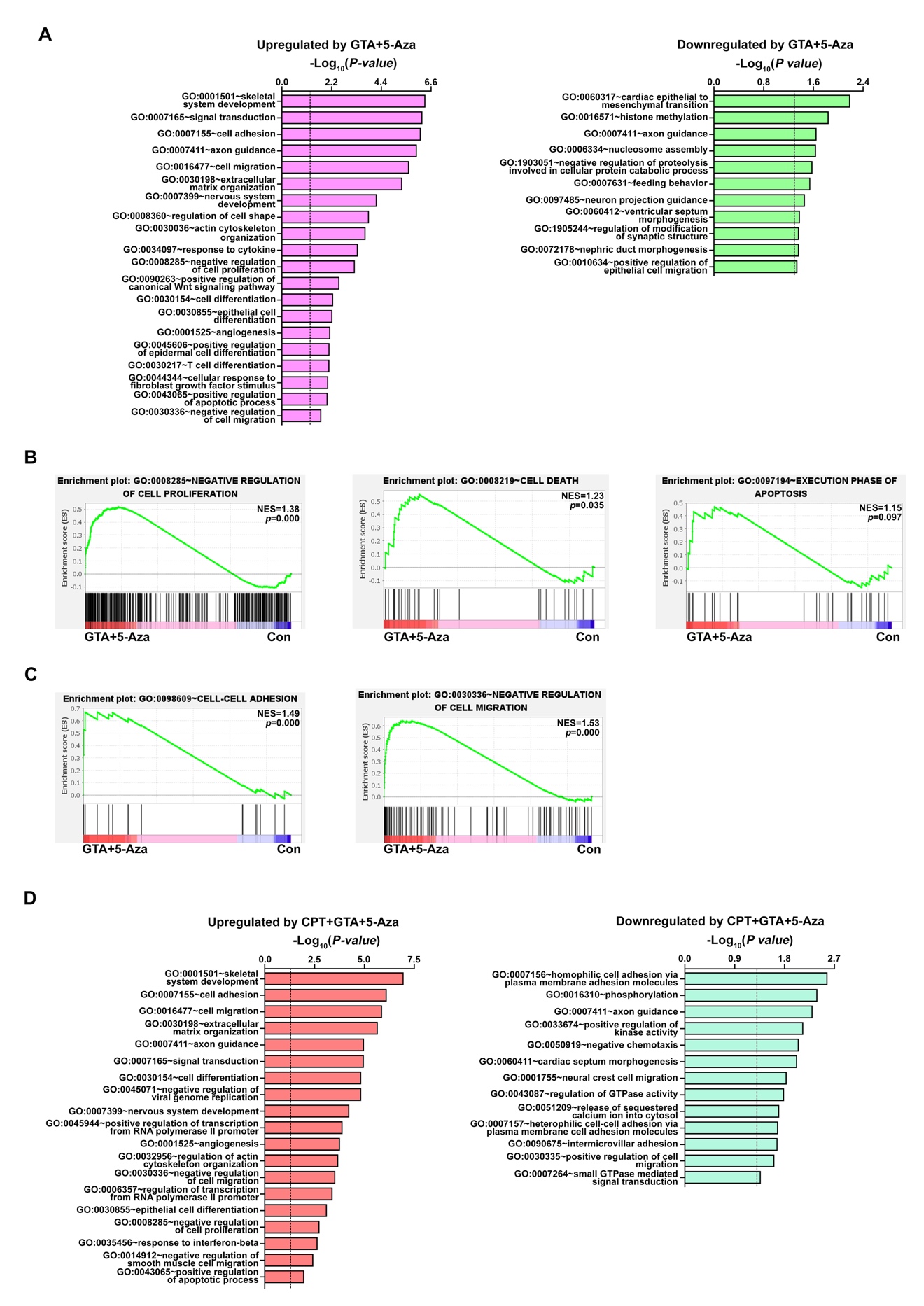
**Supplementary Fig. 8. Epigenetic priming with GTA and 5-Aza reprograms the transcriptional signature of PDAC cells.** (A) GO analysis of up-regulated genes (left) and down-regulated genes (right) by GTA+5Aza vs Con. (B & C) GSEA analysis of genes involved in cell viability (B) and cell migration (C). (D) GO analysis of up-regulated genes (left) and down-regulated genes (right) by CPT+GTA+5Aza vs Con. The dashed line indicates that *P* = 0.05.


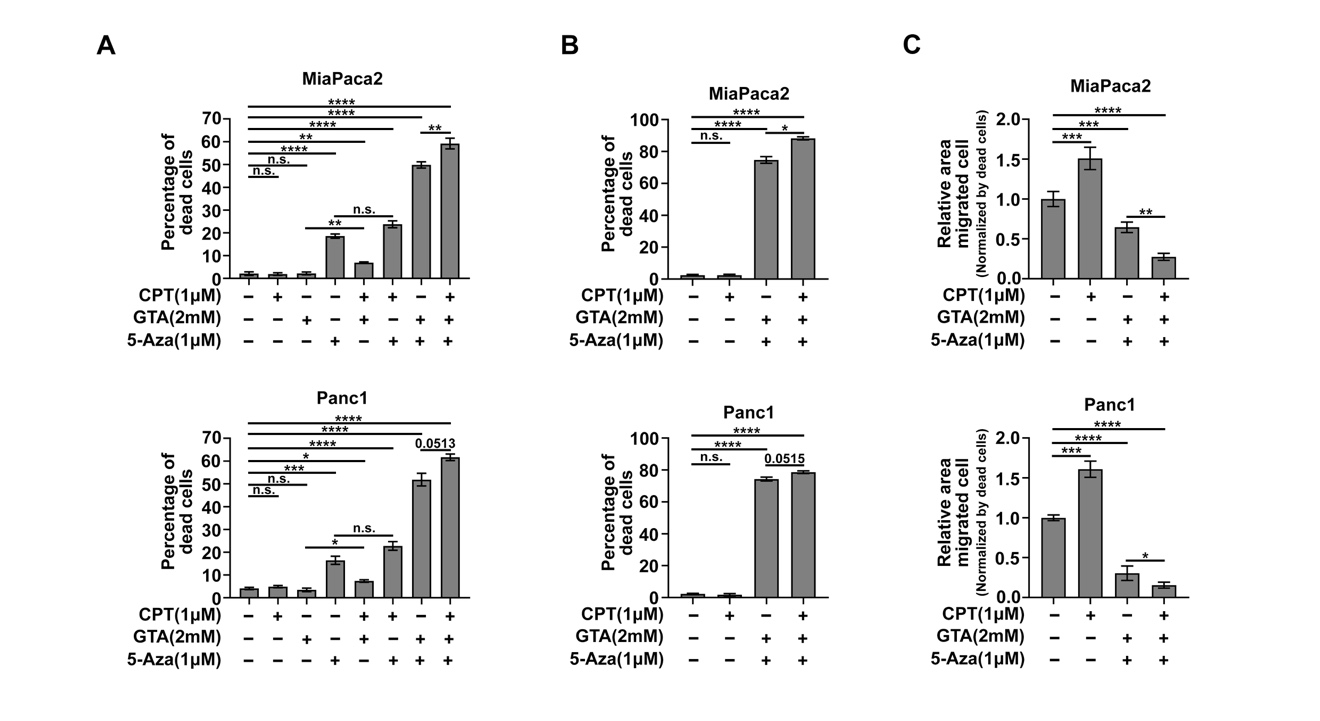


**Supplementary Fig.9. Epigenetic priming with GTA and 5-Aza enhances anti-tumor activity of Vitamin D partially through inducing cell death.** (A) Cell death of MiaPaca2 and Panc1 cells in 72-hour proliferation assay experiment. (B) Pretreatment with GTA and 5-Aza induced cell death in PDAC cells. Cells were pretreated as indicated for 24 h. (C) Cell migration was normalized by cell survival. All data are plotted as Mean ± S.E.M. Statistical significance was determined by one-way ANOVA with Tukey’s multiple comparisons test. n.s. no significance or P > 0.05; *P < 0.05; **P < 0.01; ***P < 0.005 and ****P < 0.001.


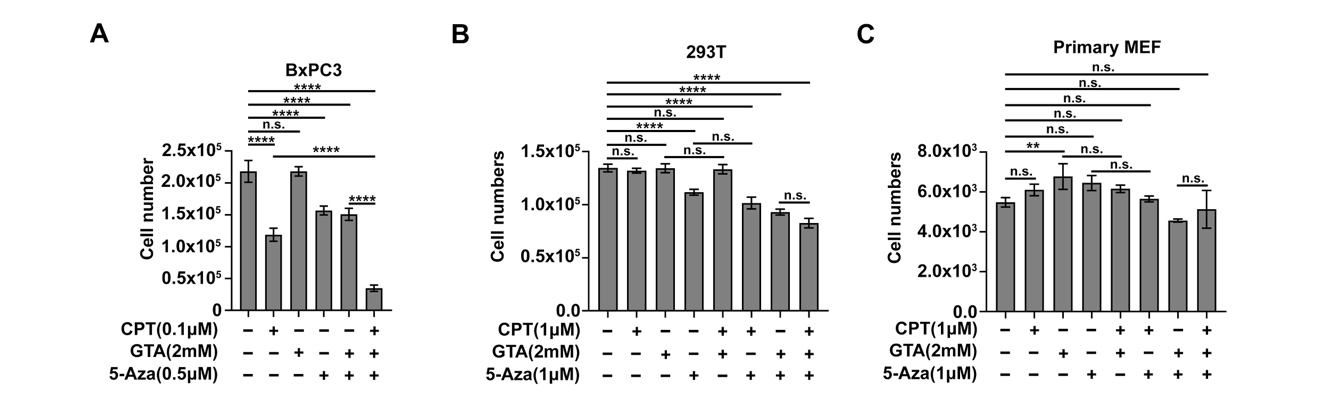


**Supplementary Fig.10. 72-hour proliferation assay for BxPC3 cells (A), 293T cells (B) and primary MEF cells (C) with indicated treatment.** All data are plotted as Mean ± S.E.M. Statistical significance was determined by one-way ANOVA with Tukey’s multiple comparisons test. n.s. no significance or P > 0.05; *P < 0.05; **P < 0.01; ***P < 0.005 and ****P < 0.001.


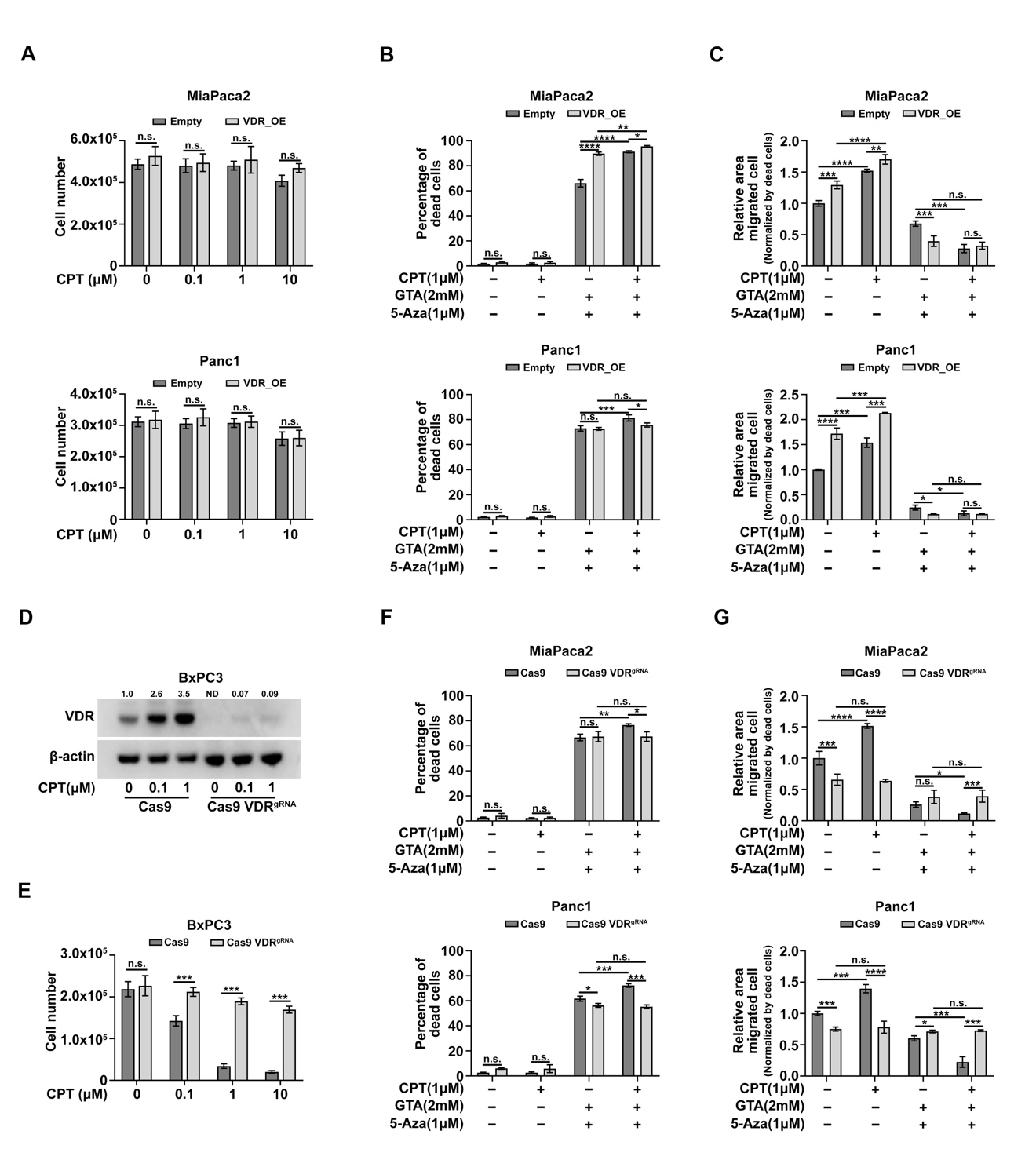


**Supplementary Fig.11. The role of VDR on the anti-tumor activity of Vitamin D in human PDAC cells.** (A) 72-hour proliferation assay showed that VDR overexpression does not trigger the anti-proliferation capacity of Vitamin D in MiaPaca2 and Panc1 cells. (B) Pretreatment with GTA and 5-Aza induced cell death in PDAC cells with or without VDR overexpression. Cells were pretreated as indicated for 24 h. (C) Cell migration was normalized by cell survival. (D) Immunoblot analysis of whole cell lysates from Cas9 and Cas9-VDR^gRNA^ transfected BxPC3 cells with indicated treatment. (E) 72-hour proliferation assay shows that VDR loss disrupts the anti-proliferation capacity of Vitamin D in BxPC3 cells. (F) Pretreatment with GTA and 5-Aza induced cell death in PDAC cells with or without *vdr* loss. Cells were pretreated as indicated for 24 h. (G) Cell migration was normalized by cell survival. ND: not detected or not determined. All data are plotted as Mean ± S.E.M. Statistical significance was determined by two-way ANOVA with Sidak’s multiple comparisons test. n.s. no significance or P > 0.05; *P < 0.05; **P < 0.01; ***P < 0.005 and ****P < 0.001.


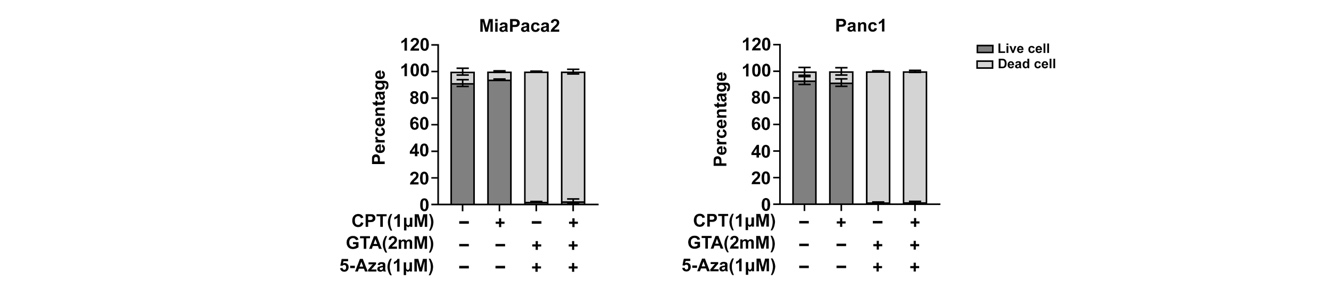


**Supplementary Fig.12. PDAC cells are vulnerable to epigenetic priming under serum-free 3D culture system.**
