# Supplementary material for: Epigenetic priming targets tumor heterogeneity to shift transcriptomic phenotype of pancreatic ductal adenocarcinoma towards a Vitamin D susceptible state
Source: Cell Death Dis. 2024 Jan 26;15(1):89. doi: 10.1038/s41419-024-06460-9 (PMC10810848; doi:10.1038/s41419-024-06460-9)
Supplement: Supplementary file 2 — original data [file 41419_2024_6460_MOESM2_ESM.pdf]

Original Data for Western blot

Fig. 1B MiaPaca2

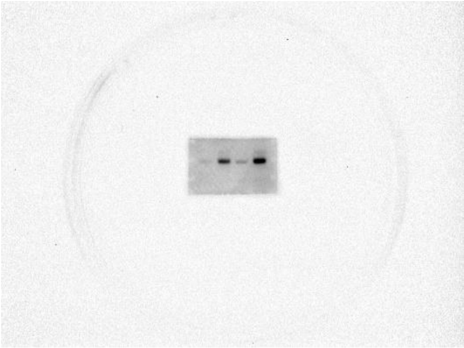
VDR

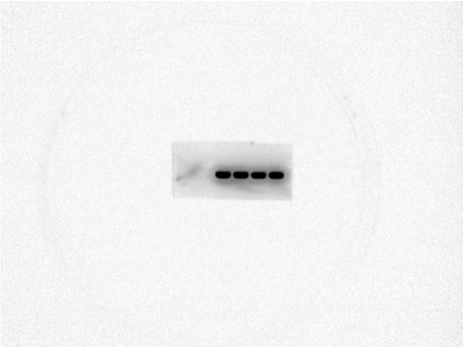
β-actin

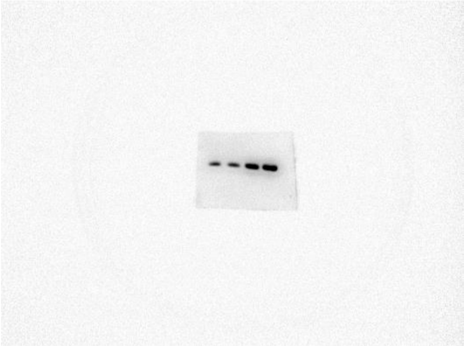
H3K27ac

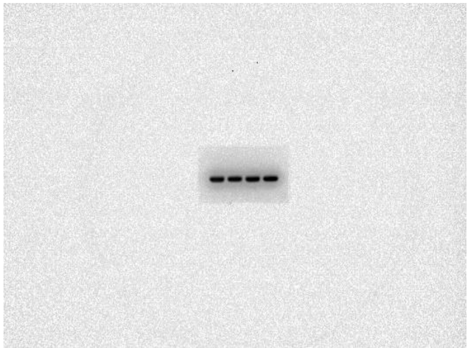
H3

Fig 1 B Panc1

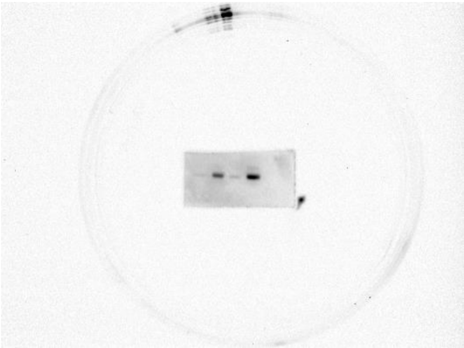
VDR

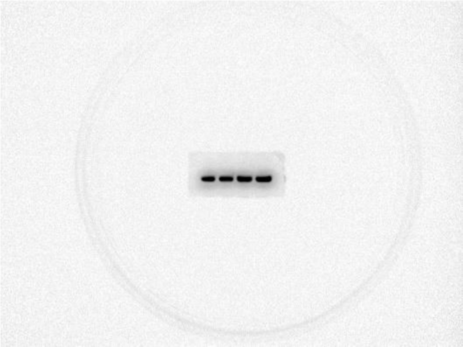
β-actin

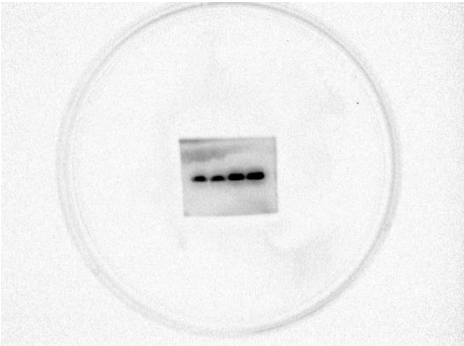
H3K27ac

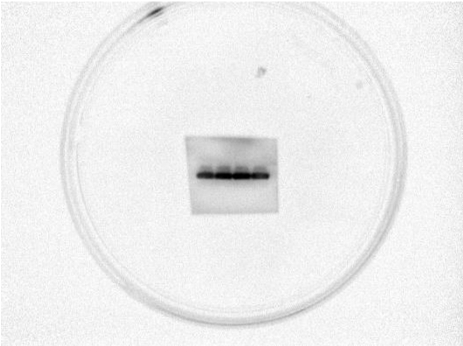
H3

Fig 1 D MiaPaca2

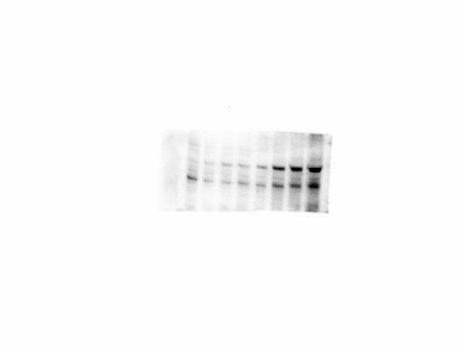

VDR

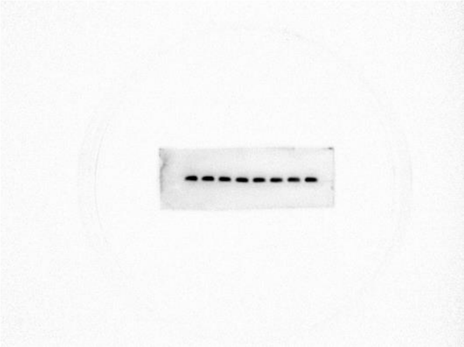

β-actin

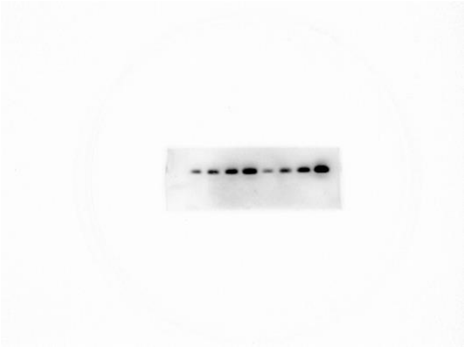

H3K27ac

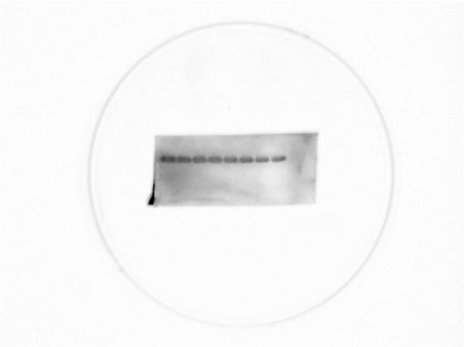

H3

Fig 1 D Panc1

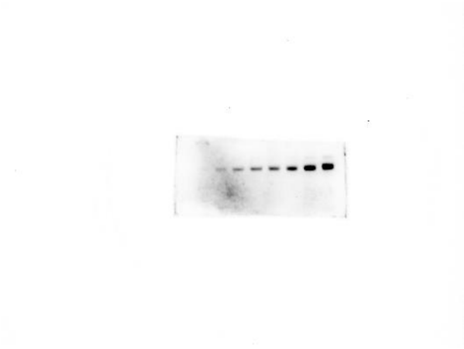

VDR

β-actin

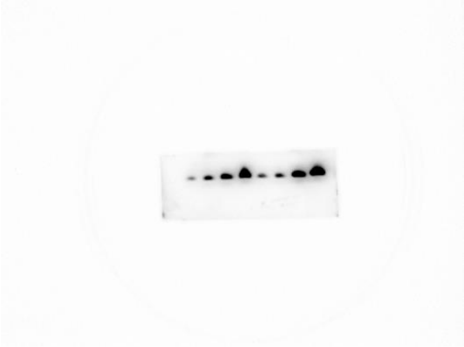

H3K27ac

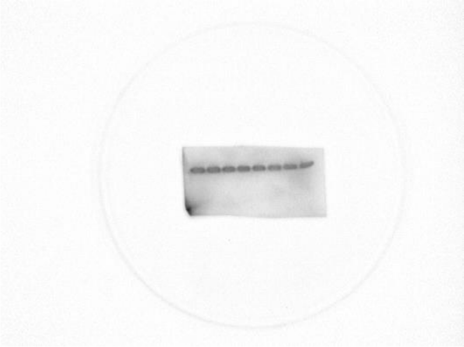

H3

Fig. 2B MiaPaca2

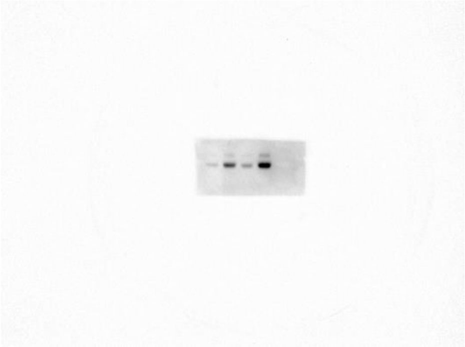

VDR

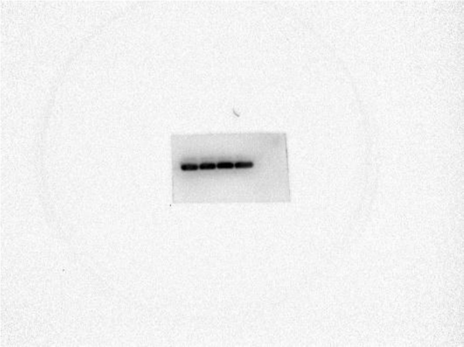

β-actin

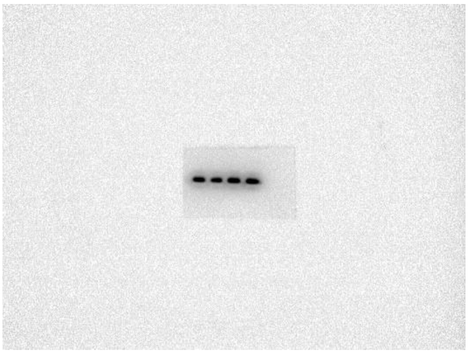

H3K27ac

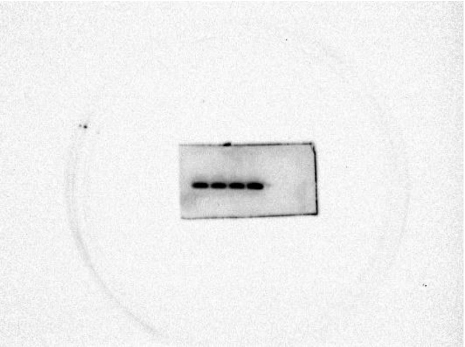

H3

Fig. 2B Panc1

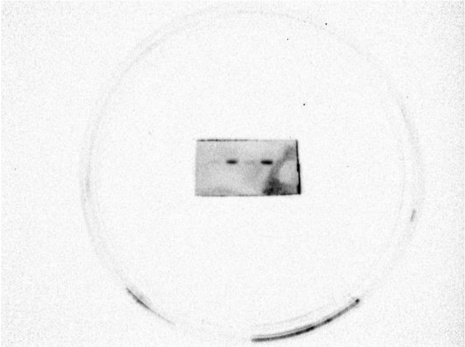

VDR

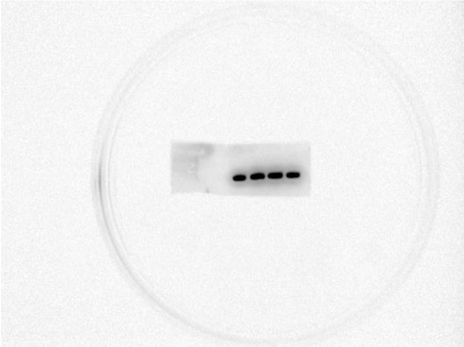

β-actin

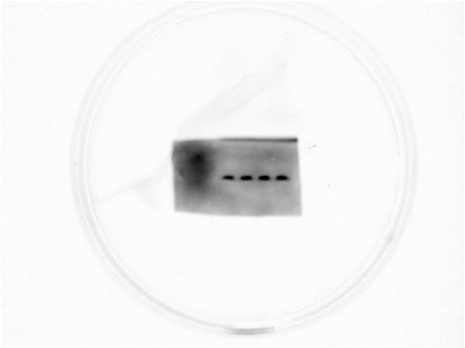

H3K27ac

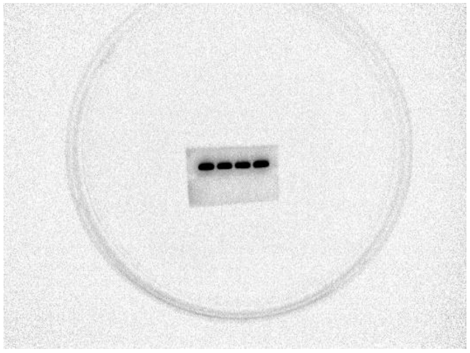

H3

Fig. 2D MiaPaca2

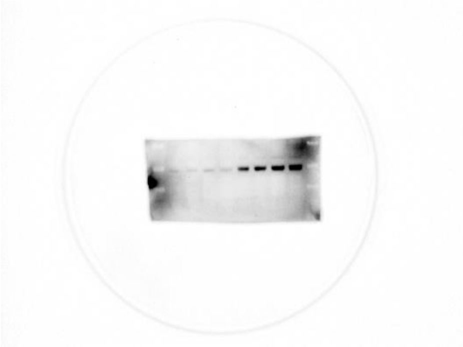
VDR

β-actin

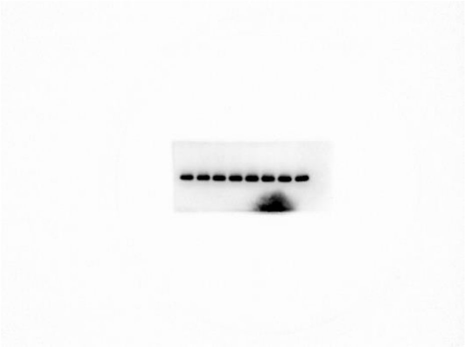
H3K27ac

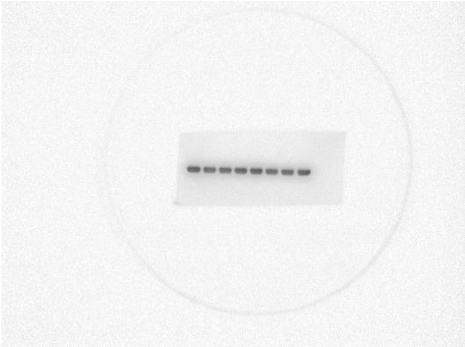
H3

Fig. 2D Panc1

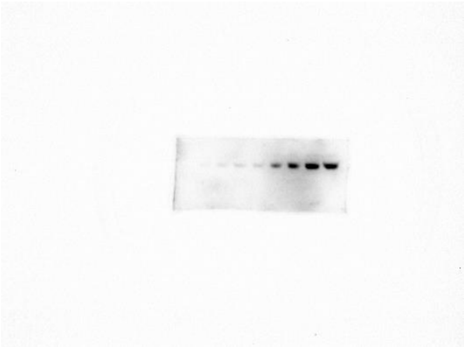
VDR

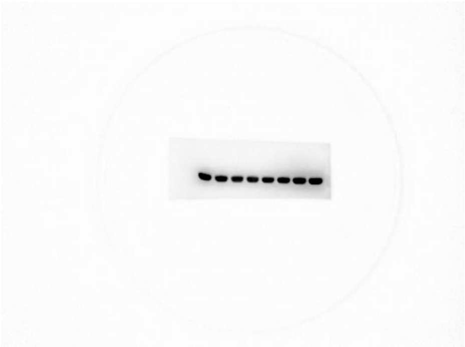
β-actin

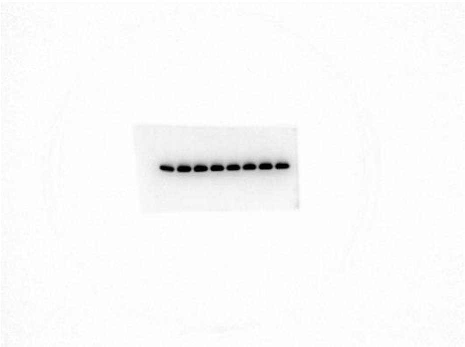
H3K27ac

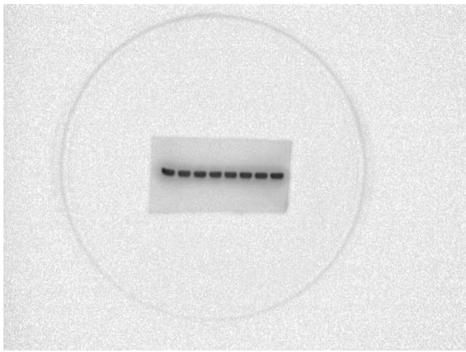
H3

Fig. 3 A MiaPaca2

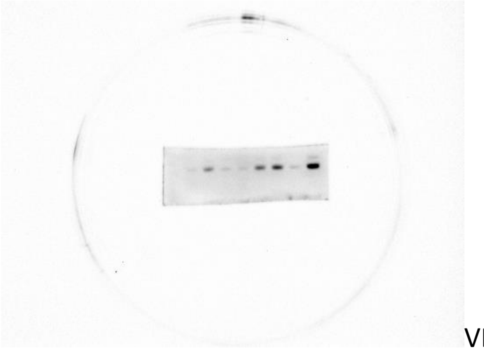
VDR

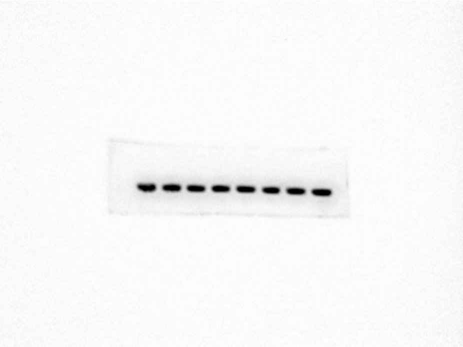
β-actin

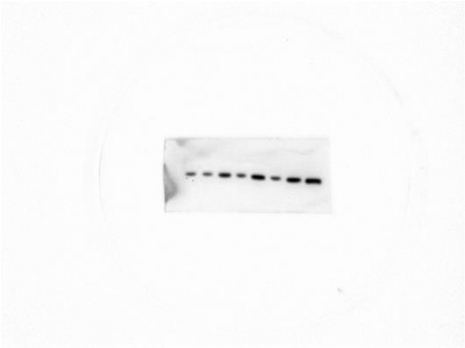
H3K27ac

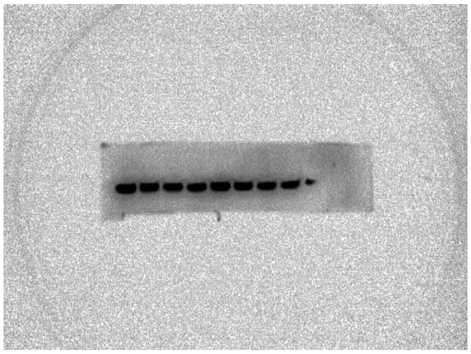
H3

Fig. 3 A Panc1

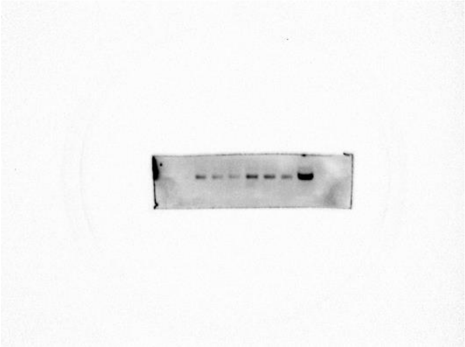
VDR

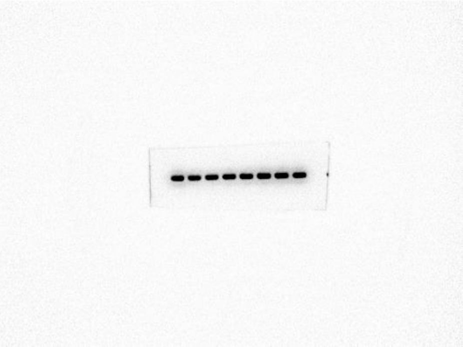
β-actin

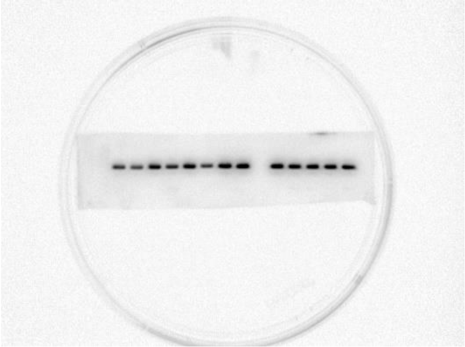
H3K27ac (Left part)

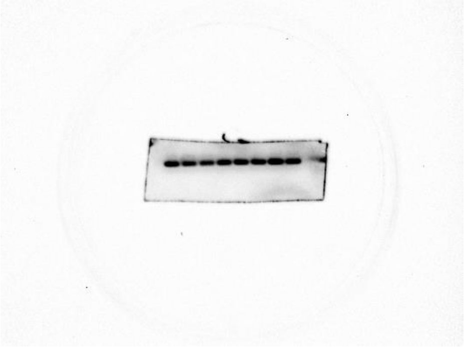
H3

Fig. 5B

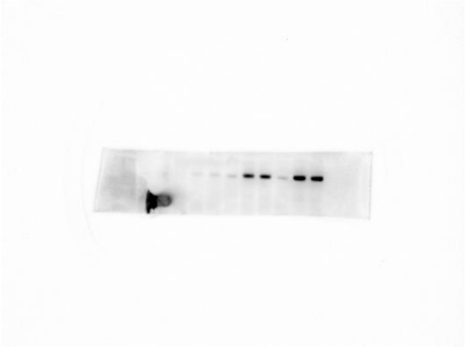

VDR (short exposure)

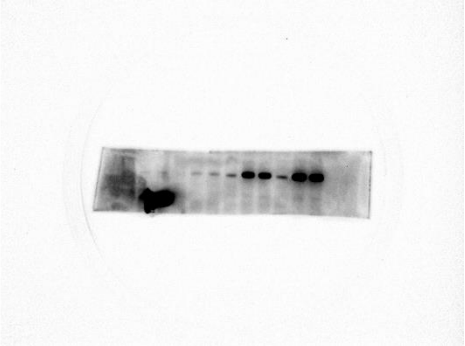

VDR(long exposure)

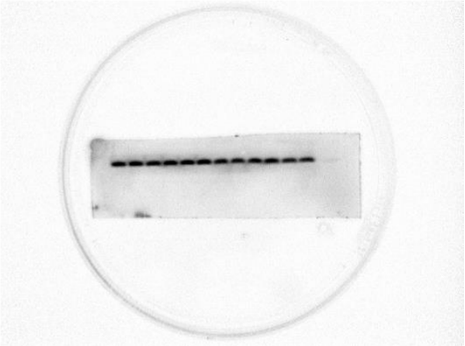

β-actin

Fig 5C BF1987

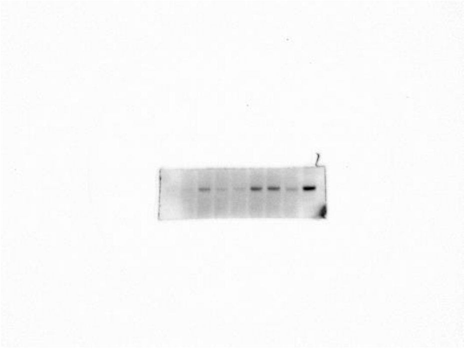
VDR

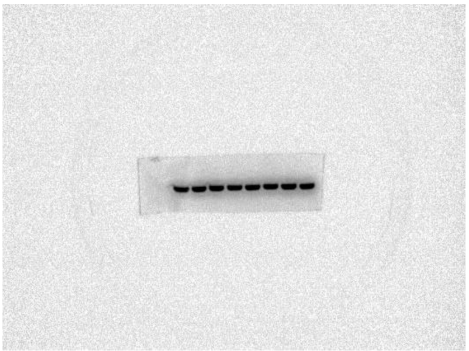
β-actin

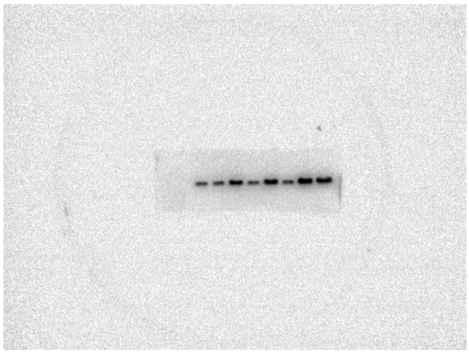
H3K27ac

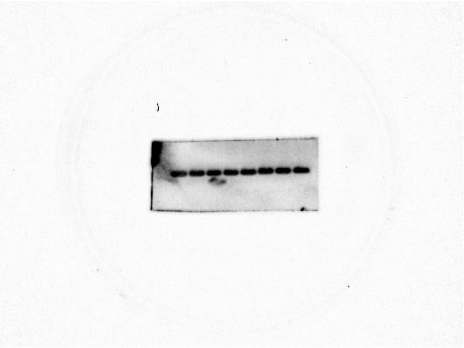
H3

Fig 5C BF2014

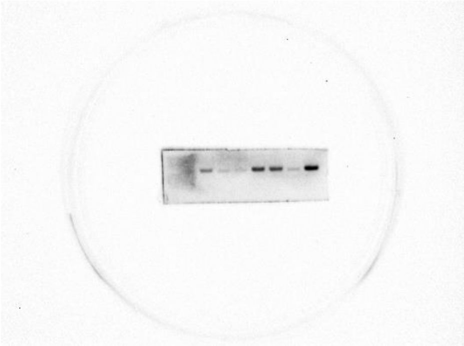
VDR

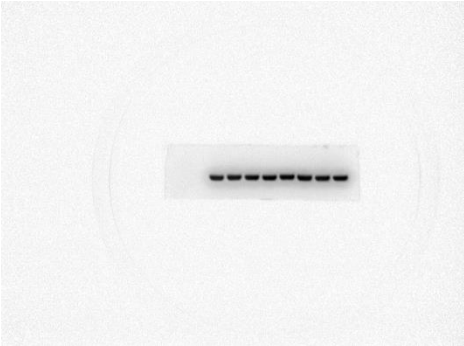
β-actin

H3K27ac

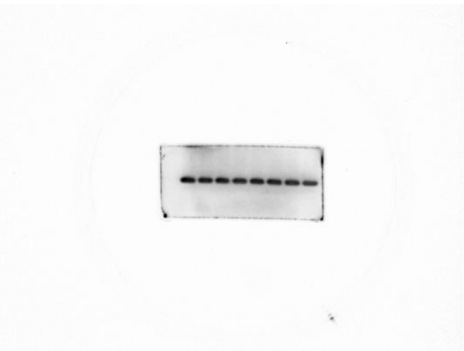
H3

Fig 6A MiaPaca2

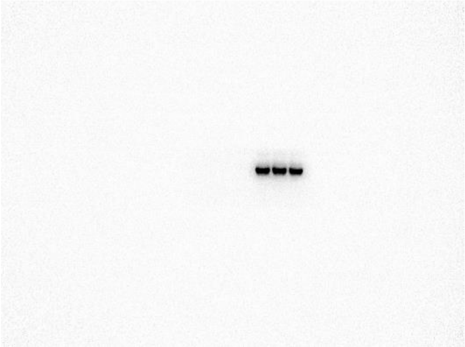

VDR (Cyt)

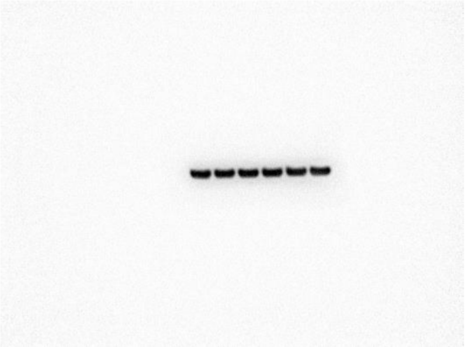

β-actin

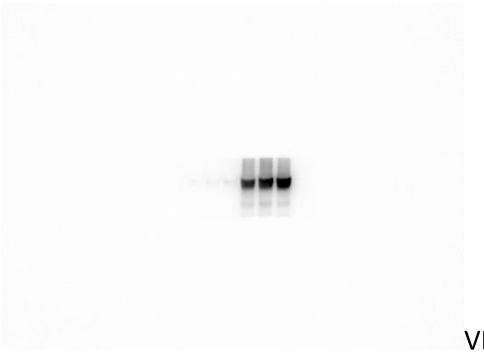

VDR(Nuclear)

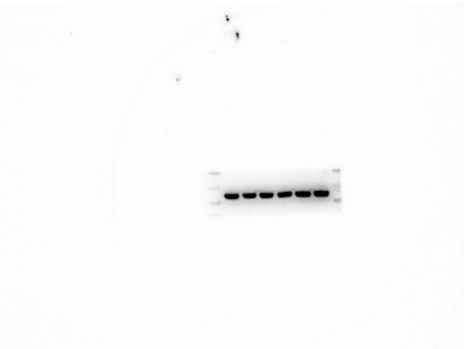

H3

Fig 6A Panc1

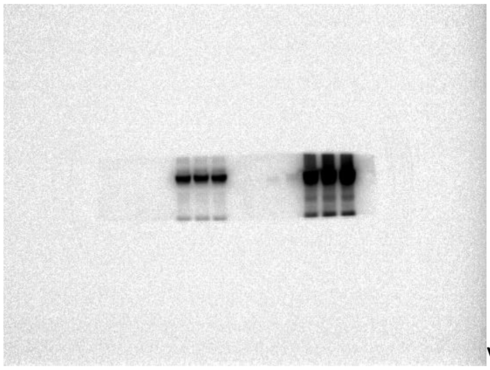

VDR (Cyt. Left)

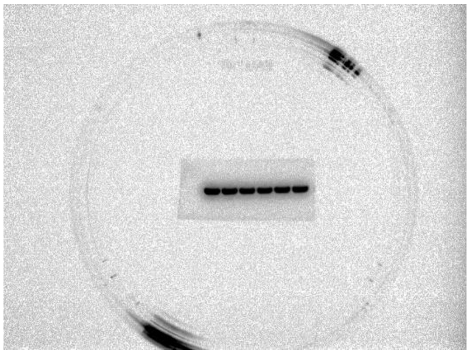

β-actin

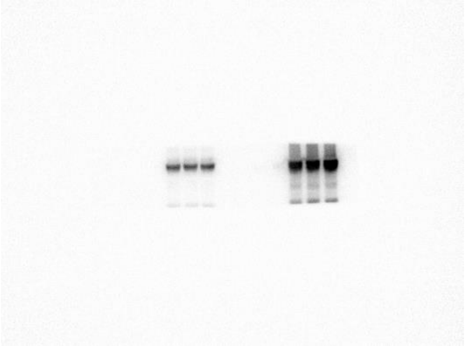

VDR(Nuc Right)

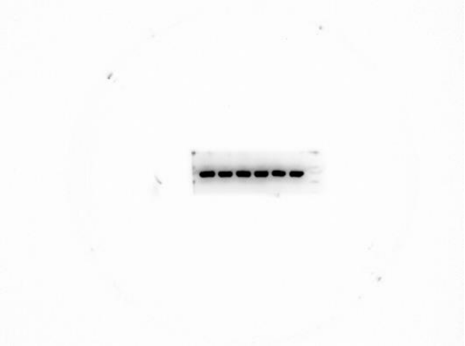

H3

Fig. 6E MiaPaca2

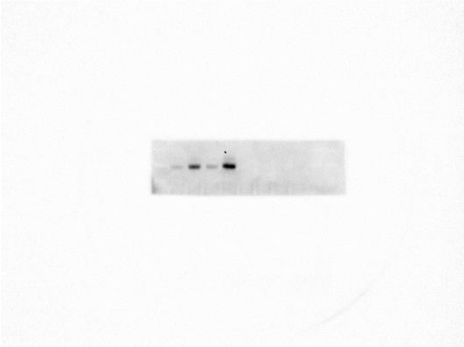

VDR

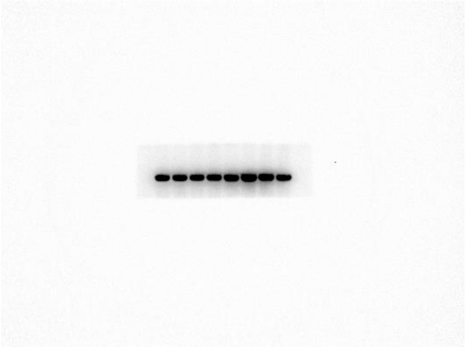

β-actin

H3K27ac

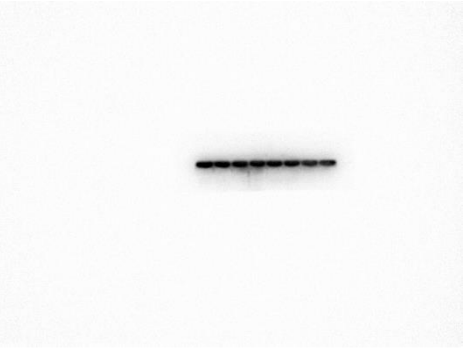

H3

Fig. 6E Panc1

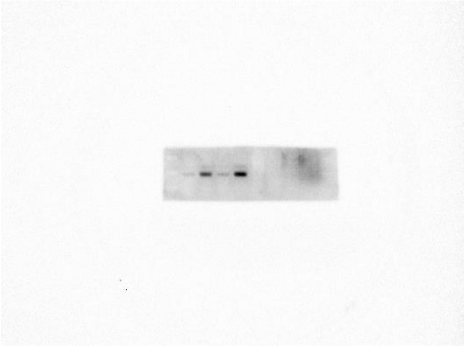

VDR

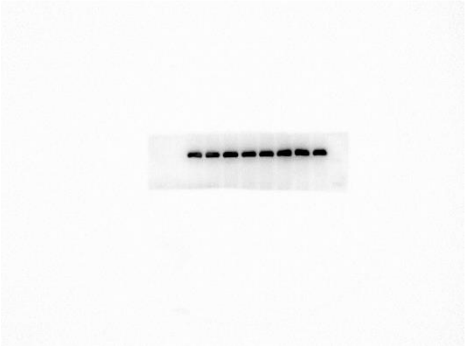

β-actin

H3K27ac

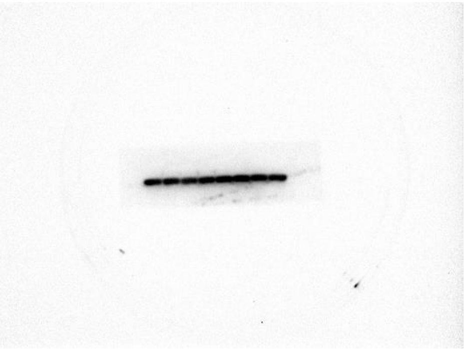

H3

Fig. S1B

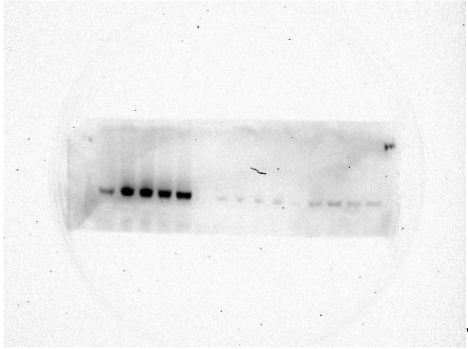
VDR (Nuc)

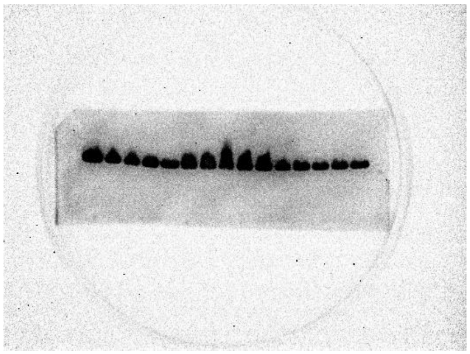
H3

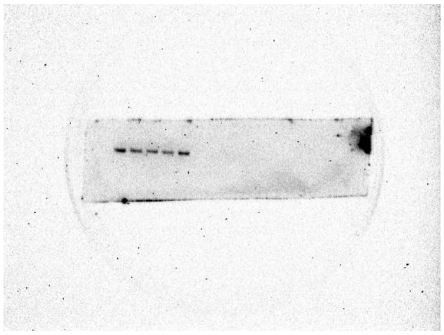
VDR (Cyt)

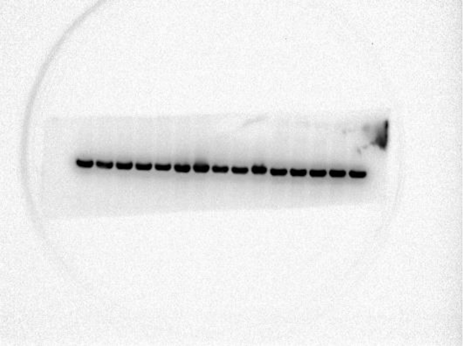
β-actin

Fig. S2B MiaPaca2

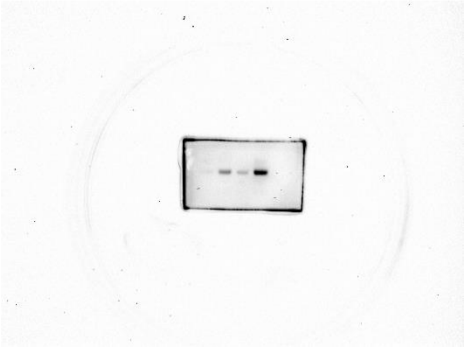
VDR

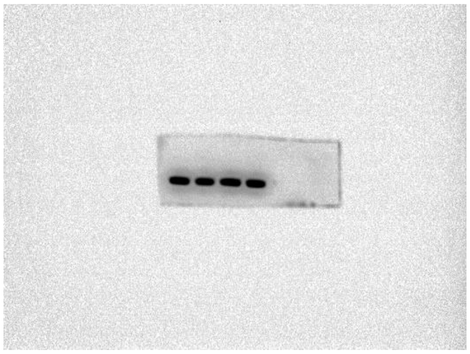
β-actin

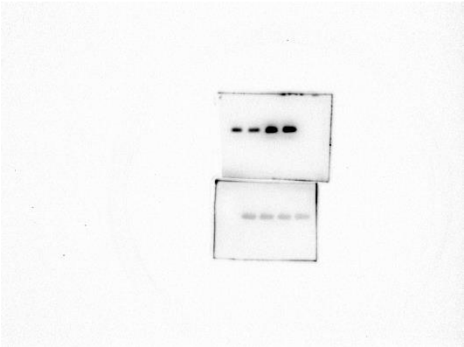
H3K27ac (Up)

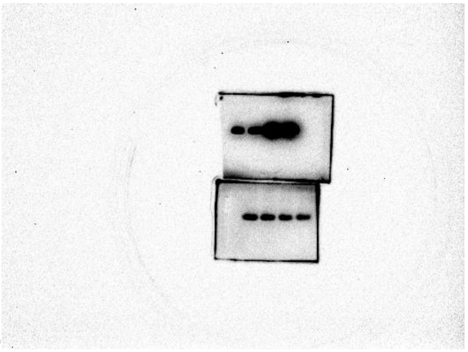
H3 (Bottom)

Fig. S2B Panc1

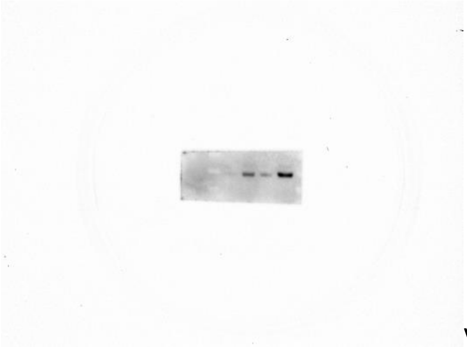
VDR

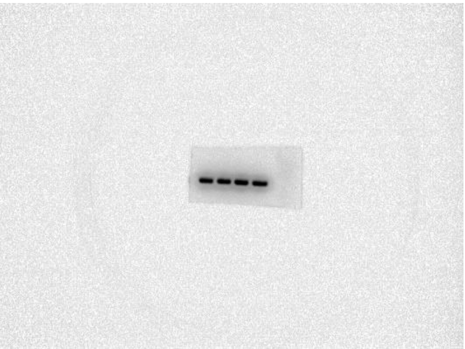
β-actin

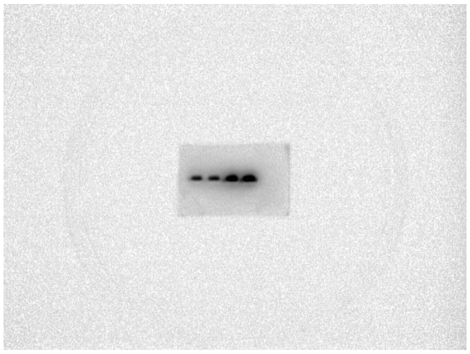
H3K27ac

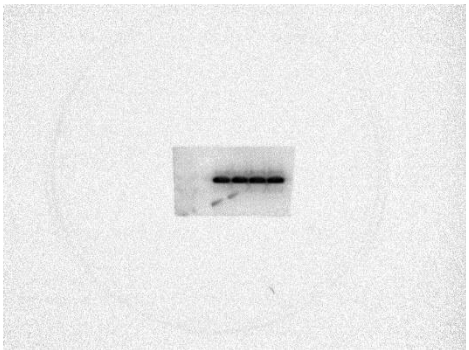
H3

Fig. S2E

H3K4ac

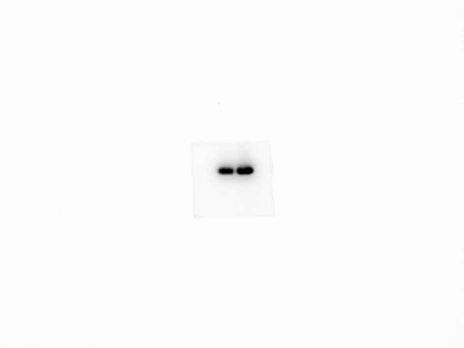
H3K9ac

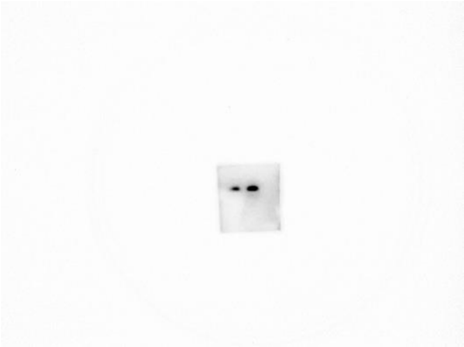
H3K27ac

H3ac

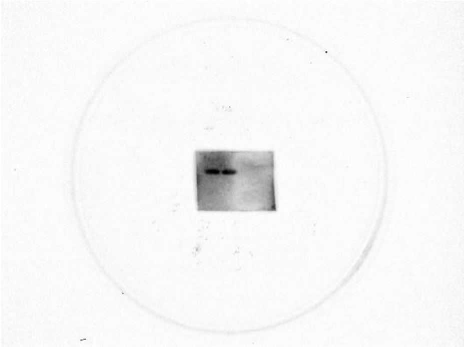

H3

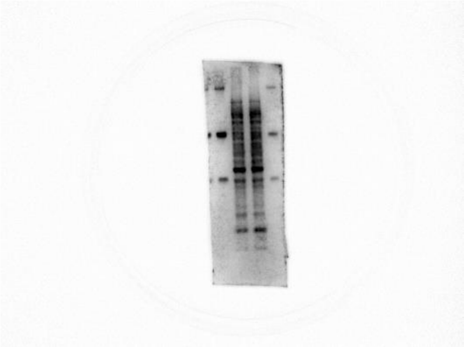

Total Kac

β-actin

Fig. S11D

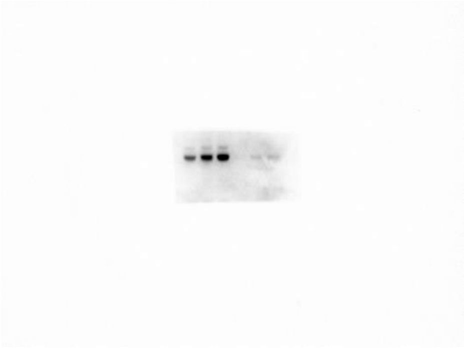

VDR

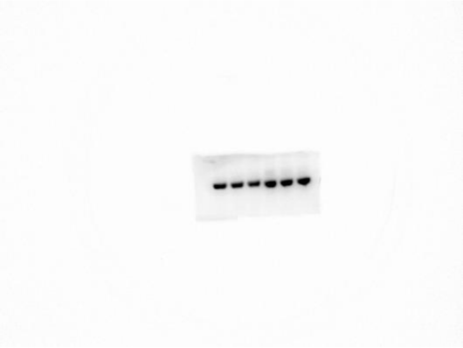

β-actin
